# Intranigral Administration of *β*-Sitosterol-*β*-D-Glucoside Elicits Neurotoxic A1 Astrocyte Reactivity and Chronic Neuroinflammation in the Rat Substantia Nigra

**DOI:** 10.1155/2020/5907591

**Published:** 2020-11-16

**Authors:** Claudia Luna-Herrera, Irma A. Martínez-Dávila, Luis O. Soto-Rojas, Yazmin M. Flores-Martinez, Manuel A. Fernandez-Parrilla, Jose Ayala-Davila, Bertha A. León-Chavez, Guadalupe Soto-Rodriguez, Victor M. Blanco-Alvarez, Francisco E. Lopez-Salas, Maria E. Gutierrez-Castillo, Bismark Gatica-Garcia, America Padilla-Viveros, Cecilia Bañuelos, David Reyes-Corona, Armando J. Espadas-Alvarez, Linda Garcés-Ramírez, Oriana Hidalgo-Alegria, Fidel De La Cruz-lópez, Daniel Martinez-Fong

**Affiliations:** ^1^Departamento de Fisiología, Escuela Nacional de Ciencias Biológicas, Instituto Politécnico Nacional, Wilfrido Massieu y Cda. Manuel Stampa s/n, C.P. 07738 Ciudad de México, Mexico; ^2^Departamento de Fisiología, Biofísica y Neurociencias, CINVESTAV, Av. Instituto Politécnico Nacional No. 2508, C.P. 07360 Ciudad de México, San Pedro Zacatenco, Mexico; ^3^Facultad de Estudios Superiores Iztacala, Universidad Nacional Autónoma de México, Av. De los Barrios No. 1, Tlalnepantla, C.P. 54090 Edo. De México, Mexico; ^4^Programa Institucional de Biomedicina Molecular, Escuela Nacional de Medicina y Homeopatía, Instituto Politécnico Nacional, Guillermo Massieu Helguera 239, C.P. 07320 Ciudad de México, Mexico; ^5^Facultad de Ciencias Químicas, Benemérita Universidad Autónoma de Puebla, Av.14 Sur y Av. San Claudio, Cd. Universitaria, Puebla, C.P. 72570 Puebla, Mexico; ^6^Facultad de Medicina, Benemérita Universidad Autónoma de Puebla, 13 Sur 2702, Puebla, C.P. 72420 Puebla, Mexico; ^7^Facultad de Enfermería, Benemérita Universidad Autónoma de Puebla, Av. 25 Poniente 1304, Los Volcanes, Puebla, C.P. 72410 Puebla, Mexico; ^8^Departamento de Biociencias e Ingeniería, Centro Interdisciplinario de Investigaciones y Estudios sobre Medio Ambiente y Desarrollo, Instituto Politécnico Nacional, 30 de Junio de 1520 s/n, C.P. 07340 Ciudad de México, Mexico; ^9^Coordinación General de Programas Multidisciplinarios, Programa Transdisciplinario en Desarrollo Científico y Tecnológico para la Sociedad, Centro de Investigación y de Estudios Avanzados, Av. Instituto Politécnico Nacional No. 2508, C.P. 07360 Ciudad de México, Mexico; ^10^Programa de Nanociencias y Nanotecnología, CINVESTAV, Av. Instituto Politécnico Nacional No. 2508, C.P. 07360 Ciudad de México, San Pedro Zacatenco, Mexico

## Abstract

Chronic consumption of *β*-sitosterol-*β*-D-glucoside (BSSG), a neurotoxin contained in cycad seeds, leads to Parkinson's disease in humans and rodents. Here, we explored whether a single intranigral administration of BSSG triggers neuroinflammation and neurotoxic A1 reactive astrocytes besides dopaminergic neurodegeneration. We injected 6 *μ*g BSSG/1 *μ*L DMSO or vehicle into the left *substantia nigra* and immunostained with antibodies against tyrosine hydroxylase (TH) together with markers of microglia (OX42), astrocytes (GFAP, S100*β*, C3), and leukocytes (CD45). We also measured nitric oxide (NO), lipid peroxidation (LPX), and proinflammatory cytokines (TNF-*α*, IL-1*β*, IL-6). The Evans blue assay was used to explore the blood-brain barrier (BBB) permeability. We found that BSSG activates NO production on days 15 and 30 and LPX on day 120. Throughout the study, high levels of TNF-*α* were present in BSSG-treated animals, whereas IL-1*β* was induced until day 60 and IL-6 until day 30. Immunoreactivity of activated microglia (899.0 ± 80.20%) and reactive astrocytes (651.50 ± 11.28%) progressively increased until day 30 and then decreased to remain 251.2 ± 48.8% (microglia) and 91.02 ± 39.8 (astrocytes) higher over controls on day 120. C3(+) cells were also GFAP and S100*β* immunoreactive, showing they were neurotoxic A1 reactive astrocytes. BBB remained permeable until day 15 when immune cell infiltration was maximum. TH immunoreactivity progressively declined, reaching 83.6 ± 1.8% reduction on day 120. Our data show that BSSG acute administration causes chronic neuroinflammation mediated by activated microglia, neurotoxic A1 reactive astrocytes, and infiltrated immune cells. The severe neuroinflammation might trigger Parkinson's disease in BSSG intoxication.

## 1. Introduction

Clinical studies indicate that neuroinflammation plays a pivotal role in Parkinson's disease (PD) [1], the second more common chronic neurodegenerative illness worldwide [2]. Postmortem studies have demonstrated the presence of activated microglia and reactive astrocytes, the professional immune cells of the central nervous system, in the brain of patients with PD. Activated microglia have been evidenced by the increased number of OX42 immunoreactive cells with a phagocytic phenotype [3]. Besides, inflammation mediators of microglial origin, such as nitric oxide (NO) and proinflammatory cytokines, have been found in mesencephalon slices, spinal cord fluid (SCF), and serum of PD patients [4–8]. Similarly, reactive astrocytes have been evidenced by the increased number of calcium-binding protein S100*β* immunoreactive cells in PD patients' brains and high levels of S100*β* in the SCF [9–11]. Different from the glial fibrillary acidic protein (GFAP), S100*β* is a more suitable neuroinflammation marker because this protein can act as a cytokine. It can be secreted and stimulates the expression of inducible nitric oxide synthase (iNOS), thus elevating NO production [12, 13]. NO is known to be involved in neuroinflammation and the subsequent degeneration of dopaminergic neurons [14] by promoting the generation of reactive oxygen species (ROS) and cyclooxygenase 2- (COX-2-) dependent synthesis of prostaglandin in microglial cells [15, 16]. A recent study has shown that the A1-classified reactive astrocytes are harmful, rapidly killing neurons and oligodendrocytes after acute central nervous system injury [10]. The A1 reactive astrocytes are induced by the classically activated neuroinflammatory microglia and identified by their immunoreactivity to complement component C3 [10].

The presence of neurotoxic A1 reactive astrocytes in PD patient brains suggests that these cells contribute to the death of dopaminergic neurons [10]. This is why the new antiparkinsonian therapy is aimed at inhibiting A1 reactive astrocytes [17, 18]. The mechanism by which neurotoxic A1 reactive astrocytes cause neuron death is still under research.

Pathological *α*-synuclein aggregates can elicit neuroinflammation in PD by activating microglia to produce ROS [19–22] and recruiting peripheral immune cells [23, 24]. T lymphocytes extravasate into the central nervous system (CNS) via a blood-brain barrier (BBB) leaky in PD patients [25]. Accordingly, large numbers of CD4(+) and CD8(+) T cells populate the ventral midbrain of patients and animal models of PD [26–28]. Recent studies indicate that the infiltrated T cells could generate an autoimmune response to *α*-synuclein [29], thus worsening and prolonging the primary neuroinflammation caused by the activated microglia. Regardless of the specific mechanism, activated microglia is the leading player in the *α*-synuclein-induced neuroinflammation. Therefore, microglia-activated A1 reactive astrocytes could also mediate the neurotoxic effect of pathological *α*-synuclein [17]. The study of this issue in other rat models of *α*-synucleinopathy is necessary to gain insight into PD pathology and validate new therapies.

Toxins present in the flour of washed seeds from the plant *Cycas micronesica* (cycad) have been linked to the amyotrophic lateral sclerosis/parkinsonism/dementia complex (ALS/PDC) in the Chamorro population of Guam island [30, 31] and have been used to generate PD-like disorders in rodents [32]. Sprague-Dawley rats fed with cycad flour for at least 16 weeks show a loss of dopaminergic neurons in the *substantia nigra pars compacta* (SNpc) and *α*-synuclein aggregates in the SNpc and striatum along with motor deficits [33].

Moreover, a faithful model for PD was developed in *Sprague-Dawley* rats chronically fed with pellets supplemented with *β*-sitosterol-D-glucoside (BSSG), a neurotoxin isolated from cycad [34, 35]. This model replicates the three cardinal features of PD, i.e., motor and cognitive dysfunctions, dopaminergic neurodegeneration, and insoluble *α*-synuclein aggregates that follow the Braak stages of PD [34]. Neuroinflammation also occurs in the chronic oral BSSG administration, as shown by a significant elevation in the number of activated microglia in the SNpc [34, 36]. However, whether BSSG administration could lead to reactive astrocyte induction, leukocyte infiltration, and production of chemical mediators of inflammation is still unknown. We recently showed that a single intranigral administration of BSSG reproduces, in less time, most of the features of oral administration, including dopaminergic neuron loss and pathological *α*-synuclein aggregation in the SNpc [37, 38].

Herein, we aim at demonstrating the induction of neurotoxic A1 reactive astrocytes as part of the inflammatory response and their link to nigral dopaminergic neurodegeneration after the stereotaxic administration of 6 *μ*g BSSG/1 *μ*L DMSO [37]. Our results confirm the activation of microglial cells and advance the knowledge showing the production of NO and proinflammatory cytokines, released by microglia, astrocytes, infiltrating leukocytes, neurons [39], and possibly by BBB-endothelial cells known to express IL-1*β* and IL-6 genes [40, 41]. We showed, for the first time, astrocyte reactivity through increasing immunoreactivity to S100*β* and C3, two specific markers of neurotoxic A1 reactive astrocytes. The increase in these markers associated with the progressive loss of TH (+) cells suggests that the neurotoxic A1 reactive astrocytes mediate the death of nigral dopaminergic neurons in the stereotaxic BSSG model in the rat. This model can be used to analyze new antiparkinsonian therapies aimed at blocking the conversion of A1 astrocytes by microglia.

## 2. Materials and Methods

### 2.1. Ethics Statement

Adult male *Wistar* rats (210-230 g) were obtained from the Laboratory of Animal Production and Experimentation Unit of CINVESTAV-IPN (Protocol 162-15). Animals were kept under standard conditions of inverted light-dark 12 h cycles in a room with a temperature of 22 ± 2°C and relative humidity of 60 ± 5%, with access to water and Chow croquettes *ad libitum*.

### 2.2. Experimental Groups

Animals (*n* = 147 in total) were randomly assigned to the BSSG group (*n* = 52), with a stereotaxic infusion of 6 *μ*g BSSG/1 *μ*L of DMSO [42], the mock group (stereotaxic injection of 1 *μ*L of DMSO; *n* = 52), and the untreated (Ut) group (without surgery and treatment; *n* = 43). Four rats of each experimental group were evaluated with nitrosative and oxidative stress assays (*n* = 4 rats per each procedure per group), three rats for ELISA (*n* = 3 rats per group), three rats for Evans blue staining (brain permeability; *n* = 3 rats per group), and three for immunostaining (*n* = 3 rats per group). These assays were performed at days 15, 30, 60, and 120 after the lesion. The total rats for the four times evaluated were 16 for nitrosative and oxidative stress assays, 12 for ELISA, and 12 for immunostaining assays. Brain permeability was evaluated at days 7, 15, 30, and 60 after the lesion (*n* = 12). For immunostaining assays, only 3 rats of the untreated group were evaluated on day 120 (*n* = 3). All the immunostaining measurements were performed in 4 anatomical levels (1 anterior, 2 medials, 1 posterior), from which mean value and SD were calculated. The total number of animals was 147, which was a minimum for statistical significance and by the experimental design in compliance with the Guide for the Care and Use of Laboratory Animals (The National Academies Collection: Reports funded by National Institutes of Health, 2011). No animal deaths occurred during the study (Supplementary Figure 1).

### 2.3. Stereotaxic Injection of BSSG

A mixture of xylazine/ketamine (10 mg/kg/100 mg/kg, i.p.) was used to anesthetize the rats and fix them with a stereotaxic apparatus. After trepanation, 1 *μ*L of BSSG (6 *μ*g/1 *μ*L of DMSO; Sigma-Aldrich; St. Louis, MO, USA) was injected into the left *substantia nigra*. The stereotaxic coordinates were anterioposterior, +3.2 mm from the interaural midpoint; mediolateral, +2.0 mm from the intraparietal suture; and dorsoventral, -6.6 mm from the dura mater [43]. A microperfusion pump (Mod. 100; Stoelting; Wood Dale, IL, USA) maintained the flow rate at 0.16 *μ*L/min. After injecting the total volume, the needle was allowed to remain in the brain for 5 min and then was withdrawn in 1 min steps to avoid reflux of the injected solution. The mock group was injected with 1 *μ*L of DMSO. After surgery, the surgical wound was sutured, and rats were maintained in an individual cage until complete recovery.

### 2.4. Dissection of Cerebral Nuclei for Molecular and Biochemical Assays

Animals were euthanized with sodium pentobarbital (50 mg/kg; intraperitoneally). For biochemical measurements (nitrosative and oxidative stress) and ELISA assays, each brain was obtained after decapitation and dissected out free of meninges and immediately submerged in cold PBS. Using a cold metallic rat brain matrix (Stoelting; Wood Dale, IL, USA), we cut twelve 0.5 mm coronal slices of each brain between the occipitotemporal anterior border and the anterior border of the cerebellum [44]. The *substantia nigra* was quickly dissected out from each coronal slice in cold and sterile conditions using a stereomicroscope (Leica ZOOM 2000; Buffalo, NY, USA) equipped with a void metallic stage to contained dry ice [44]. Each sample was immediately stored in a respective Eppendorf tube at −70°C until used [45]. For immunostaining and brain permeability, the animals were intracardially perfused with 100 mL of PBS, followed by 100 mL 4% paraformaldehyde in PBS, as previously described by Flores-Martinez et al. [44]. The brain was dissected out and maintained in the fixative for 24 h at 4°C and then in 30% sucrose in PBS at 4°C. For immunostaining assay, the brain was frozen and then sectioned at 20 *μ*m thickness using a sliding microtome (Leica SM1100; Heidelberg, Germany). The slices were consecutively collected in 6 wells, and only those in one well were used for immunostaining. For evaluation of brain permeability, the mesencephalon was cut into 100 *μ*m thick coronal slices with the aid of a vibratome (Leica Microsystems Inc, VT1200S; Buffalo Grove, IL, USA).

### 2.5. NO Production Measurements

We followed the method of Flores-Martinez et al. [44] to measure nitrite (NO_2_^−^) accumulation in the supernatant of homogenized *substantia nigra* samples as an index of nitric oxide (NO) production. Briefly, the tissue samples were mechanically homogenized in phosphate-buffered saline pH 7.4 (PBS). Homogenates were centrifuged at 20,000 g for 30 min at 4°C, and 2.5 *μ*L of supernatant was used to measure NO by adding 100 *μ*L of the Griess reagent. The color in the samples was read at 540 nm with a Nanodrop (Thermo Fisher Scientific; Wilmington, USA), and the values were interpolated in a standard curve of sodium nitrite (NaNO_2_; 1 to 10 *μ*M) to calculate the experimental nitrite values. The protein content was measured in the pellet using the BCA method and bovine serum albumin (BSA) for the standard curve following the manufacturer's protocol (Thermo Fisher Scientific; Rockford, IL, USA). The nitrite content values were expressed as *μ*mol/mg of protein.

### 2.6. Assessment of Lipid Peroxidation

We measured malondialdehyde (MDA) and 4-hydroxyalkenals (4-HAE) concentration in the supernatant of homogenized *substantia nigra* samples as an index of lipid peroxidation, following the methodology reported by Flores-Martinez et al. [44]. Briefly, the samples were homogenized in PBS and centrifuged at 20,000 g at 4°C for 40 min. Then, 100 *μ*L of the supernatant was supplemented with 325 *μ*L of a mixture of acetonitrile to methanol (3 : 1 volume) containing 10.3 mM N-methyl-2-phenylindole. The colorimetric reaction was initiated by the addition of 75 *μ*L of methanesulfonic acid. The reaction mixture was vigorously shaken and incubated at 45°C for 1 h and then centrifuged at 1000 g for 10 min. The absorbance was interpolated in a 1,1,3,3-tetramethoxypropane standard curve (0.5 to 5 *μ*M) to calculate MDA and 4-HAE content in the samples. The protein content was measured in the pellet using the BCA method and bovine serum albumin (BSA) for the standard curve following the manufacturer's protocol (Thermo Fisher Scientific; Rockford, IL, USA). The values were expressed as *μ*mol of MDA+ 4-HAE/mg of protein.

### 2.7. Immunostaining

The slices were permeabilized with PBS-0.1% Triton for 20 min and incubated with 1% BSA in PBS-0.1% Triton for 30 min to block unspecific binding sites. Then, they were incubated with the primary antibodies overnight at 4°C and with the secondary antibodies for 2 h at room temperature (RT). For immunofluorescence, the primary antibodies were a rabbit polyclonal anti-TH (1 : 1000; Merck Millipore, USA), a mouse monoclonal anti-CD11b/c (OX42; 1 : 200; Abcam, Cambridge, UK), a goat polyclonal anti-ionized calcium-binding adapter molecule 1 (Iba1) as a microglial marker (1 : 500 Abcam; Cambridge,UK), a mouse anti-CD45 (1 : 50; BD Bioscience, USA), a mouse monoclonal anti-GFAP Clone GA5 (1 : 500; Cell Signaling Technology; Danvers, Massachusetts, USA), a mouse monoclonal anti-S100*β* (1 : 200; Merck-Sigma-Aldrich, St. Louis, MO, USA), and a goat polyclonal anti-C3 (1 : 50; Invitrogen; Waltham, Massachusetts, USA). The secondary antibodies were an Alexa 488 chicken anti-rabbit H + L IgG (1 : 300; Invitrogen Molecular Probes; Eugene, OR, USA), an Alexa 488 chicken anti-goat H + L IgG (1 : 300; Invitrogen Molecular Probes; Eugene, OR, USA), and a Texas red horse anti-mouse H + L IgG (1 : 500; Vector Laboratories; Burlingame, CA, USA). After washing with PBS, the slices were mounted on glass slides using VECTASHIELD (Vector Laboratories; Burlingame, CA, USA). The fluorescence images were obtained with a Leica confocal microscope (TCS SP8; Heidelberg, Germany), using 20x, 40x, 63x, and 100x objectives. Serial 1 *μ*m optical sections were also obtained in the *Z*-series (scanning rate of 600 Hz). The images were acquired and analyzed with the LAS AF software (Leica Application Suite; Leica Microsystems; Nussloch, Germany). The immunofluorescence area density (IFAD) for the double fluorescence assays was measured using the ImageJ software v.1.46r (National Institutes of Health; Bethesda, MD). The measurements were made on images taken with a 20x (TH-GFAP), 40x (TH-OX42, TH-S100*β*, and S100*β*-GFAP), and 63x (C3-GFAP, C3-S100*β*, and TH-CD45) of the central zone of the SNpc in four different anatomic levels (one rostral, two medials, and one caudal) per rat (*n* = 3 independent rats per group and time).

Immunohistochemistry staining of microglial cells was performed in permeabilized slices incubated with a chicken polyclonal Iba1 (1 : 1000; Abcam; Cambridge, UK) overnight at 4°C, followed by incubation with a biotinylated donkey anti-chicken IgG (1 : 500; Jackson ImmunoResearch; Palo Alto, CA, USA) for 2 h at RT. Endogenous peroxidase was eliminated by incubating the slices with 3% hydrogen peroxide in PBS/Triton and 10% methanol at RT for 10 min. The immunohistochemical staining was developed using the ABC kit (1,10; Vector Laboratories; Burlingame, CA, USA) and 3′3-diaminobenzidine (DAB; Sigma-Aldrich; St. Louis, MO, USA) as reported previously [38]. The brain slices were washed 3 times for 5 min in PBS, counterstained with *β*-Gal and mounted on slides using Entellan resin (Merck, KGaA; Darmstadt, Germany), and observed with a light Leica DMIRE2 microscope with 63x (oil immersion) objective (Leica Microsystems; Nussloch, Germany).

### 2.8. Enzyme-Linked Immunosorbent Assay (ELISA)

We followed the methods of Flores-Martinez et al. [44] to measure TNF-*α*, IL-1*β*, and IL-6. Briefly, the left *substantia nigra* (*n* = 3 independent rats) was homogenized using extraction buffer containing 100 mM Tris HCl (pH 7.4), 750 mM NaCl (sodium chloride), 10 mM EDTA (ethylenediaminetetraacetic acid), 5 mM EGTA (ethylene glycol tetraacetic acid), and mix of protease inhibitors (Mini EDTA-free Protease Inhibitor Cocktail Tablets) used as indicated by the manufacturer (Roche, Basel, Switzerland) [46]. The *substantia nigra* samples were centrifuged at 1000 g for 10 minutes at 4°C. Then, the supernatant was centrifuged at 20000 g for 40 min at 4°C again to eliminate the remaining debris. The levels of inflammatory cytokines were detected by ELISA technique, using the Milliplex MAP Rat cytokine/chemokine magnetic bead panel kit (RECYTMAG_65K; Millipore; Temecula, CA, USA), and reading was done by the LUMINEX MAGPIX detection system with the xPONET software (Millipore Corporation; Billerica, MA, USA). The values in the supernatant were extrapolated in a curve of 2.4 to 10000 pg/mL for TNF-*α* and IL-1*β* and 73.2 to 300000 pg/mL for IL-6. The pellet was resuspended to measure protein content using the BCA method and bovine serum albumin (BSA) for the standard curve following the manufacturer's protocol (Thermo Fisher Scientific; Rockford, IL, USA). The values were expressed as pg of cytokine/mg of protein.

### 2.9. Evaluation of Brain Permeability

We injected a 2% Evans blue dye solution in PBS (4 mL/kg of body weight) into the caudal vein [47] of untreated, mock, and BSSG rats at days 7, 15, 30, and 60 after the BSSG injection. After 24 h, the rats were anesthetized and perfused as described in the immunostaining section. Upon completion perfusion of the fixative, the brain was dissected out, and the mesencephalon was cut into 100 *μ*m thick coronal slices with the aid of a vibratome (Leica Microsystems Inc, VT1200S; Buffalo Grove, IL, USA). Immediately, images were captured using a Leica stereomicroscope MZ6 equipped with a digital camera (Heidelberg, Germany).

### 2.10. Statistical Analysis

All values were presented as the mean ± standard deviation (SD) from at least 3 independent experiments (*n* = 3). The differences among groups were analyzed using repeated-measures two-way ANOVA and Bonferroni *post hoc* test. One-way ANOVA and Newman-Keuls *post hoc* test were used to analyze Iba1 data. The statistical analysis was made with Sigma Plot 12.0, and the graphs were built with GraphPad Prism 5.0 (GraphPad Software Inc; La Jolla, CA, USA). Statistical significance was considered at *P* < 0.05.

## 3. Results

### 3.1. Nitrosative and Oxidative Stress

**Figure 1:**
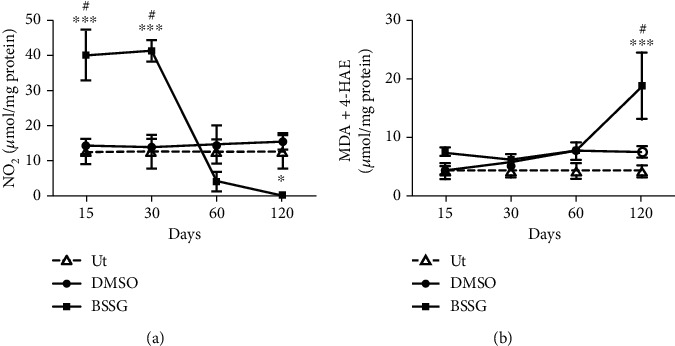
Nitrosative and lipid peroxidation after a BSSG injection in the SNpc. (a) Nitrosative stress was evaluated through the measurement of nitrite levels. (b) Lipid peroxidation was assessed as a proxy for oxidative stress by MDA and 4-HAE determinations. Ut: untreated control rats; Mock: rats injected with vehicle (DMSO); BSSG: rats injected with 6 *μ*g of BSSG. The values represent the mean ± SD from 4 rats for each time point and each experimental condition. Two-way ANOVA and Bonferroni post hoc tests were applied. (^∗∗∗^) indicates a *P* < 0.001 for statistical difference between the BSSG group compared with the Ut group or (#) for the respective mock group.

Nitrite concentration was assessed as a marker of nitrosative stress (*n* = 4 rats for each time point), while MDA and 4-HAE levels were assessed as a lipid peroxidation marker of oxidative stress (*n* = 4). Importantly, these biomarkers remained constant in the Ut and mock control groups. BSSG administration in the SNpc provoked a 4-fold increase in NO levels on days 15 and 30 postadministration as compared with the untreated and mock groups (Figure 1(a)). Afterward, NO levels decreased and remained below the untreated and mock groups until the end of the study (Figure 1(a)).

A significant 1.5-fold increase in NO levels was observed after DMSO administration only at day 120 as compared with the untreated control group (Figure 1(a)). In contrast, lipid peroxidation was not different except at day 120 after BSSG injection as compared with the untreated and mock groups (Figure 1(b)).

### 3.2. Time Course of Microglia Activation

**Figure 2:**
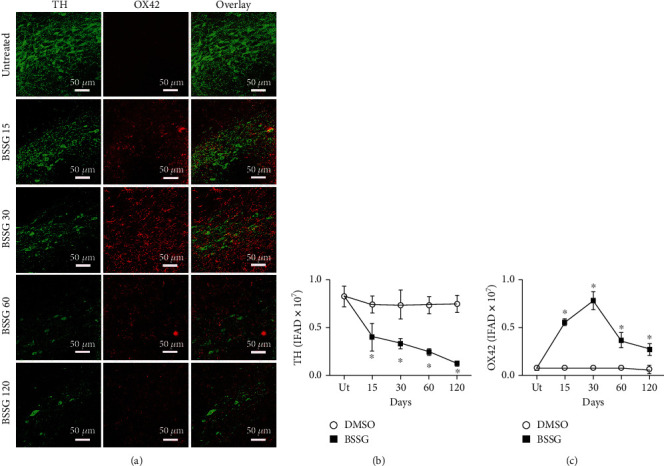
Time course of OX42 immunoreactivity after a BSSG injection in the SNpc. (a) Representative micrographs of the double immunofluorescence for TH and OX42 in untreated rats (Ut) and rats at different times (shown at the left side of micrographs) after BSSG injection. Immunofluorescence area density (IFAD) for TH (b) and OX42 (c) was determined using the ImageJ software v.1.46r (National Institutes of Health, Bethesda, MD). The TH and OX42 values for the mock rats correspond to the quantification in Supplementary Figure 2. The values are the mean ± SD from four anatomical levels. *n* = 3 independent rats in each time of each experimental condition. Two-way ANOVA and post hoc Bonferroni tests were applied for statistical analysis. (^∗^) indicates a *P* < 0.05 compared with the DMSO mock and Ut groups of the respective immunostaining.

**Figure 3:**
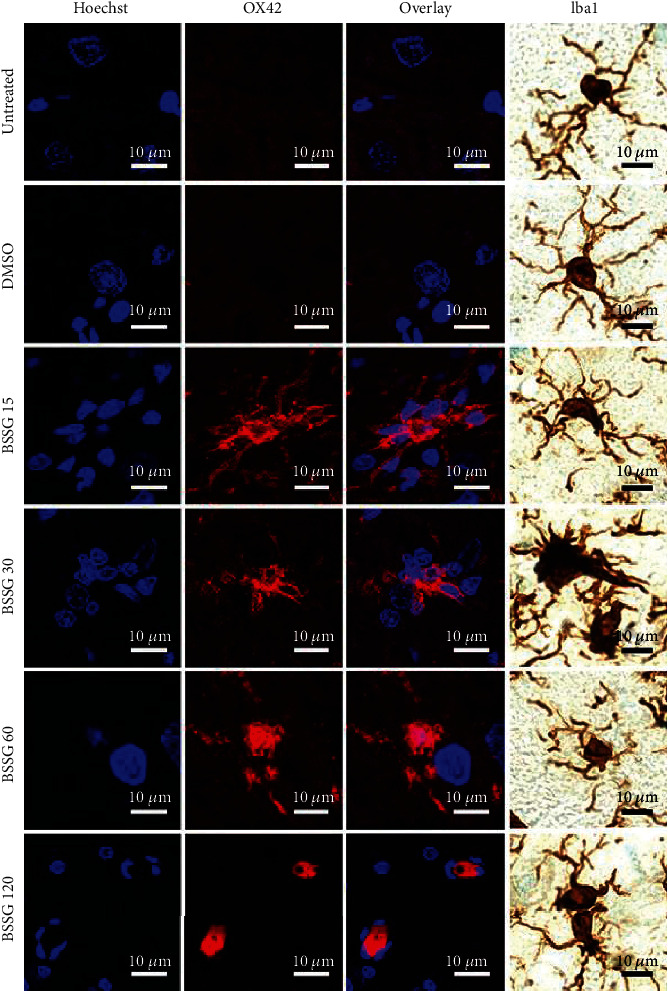
Changes in the cell form during activation of microglia in the SNpc after BSSG administration. Representative confocal micrographs of OX42(+) and Iba1(+) cells in the SNpc of untreated and DMSO mock control rats and of rats at different days post-BSSG injection (shown at the left side of the micrographs).

The double immunofluorescence assays showed that TH and OX42 immunoreactivities in the SNpc of the mock group were not statistically different from the respective untreated controls throughout the study (Figure 2 and Supplementary Figure 2). BSSG administration caused a progressive decrease of TH immunoreactivity in the SNpc, reaching an 83.6 ± 1.8% reduction on day 120 after the administration (Figures 2(a) and 2(b)). Conversely, OX42 immunoreactivity gradually increased up to 899.0 ± 80.20% over the control values on day 30 to decrease afterward and remain 251.2 ± 48.8% higher than the basal values at the end of the study (Figures 2(a) and 2(c)). Iba1 immunohistochemistry assays displayed the normal population and characteristics of microglia in the *substantia nigra* of the untreated healthy group (Supplementary Figure 3). A similar pattern in the population and morphology of Iba1(+) cells was observed in the DMSO group, confirming that this BSSG solvent did not activate microglia (Supplementary Figure 3 and Supplementary Figure 4). The opposite effect occurred in the BSSG group, where the increase in the Iba1(+) cell number over time was similar to that of OX42(+) cells (Figure 3 and Supplementary Figures 3 and 4), giving further support to microglial activation development.

Recent studies propose that changes in cell form reflect the activation state and function of microglia in acute lipopolysaccharide- (LPS-) induced neuroinflammation in the SNpc, as recently shown by Flores-Martinez et al. [44, 48]. Interestingly, BSSG-induced changes in the form of OX42-immunoreactive cells and Iba1(+) cells are similar to those induced by LPS, but they appear throughout the 120 days evaluated (Figure 3) than in the LPS model, where these changes in microglial shape occur only for 7 days [44]. OX42 and Iba1 immunoreactivity in untreated and mock conditions suggest the resting or quiescent condition of microglia (Figure 3). Robust branched cells, with long thick branches, and well-defined enlarged soma appeared on day 15 post-BSSG treatment (Figure 3). Irregular shaped OX42(+) and Iba1(+) cells with short, stout branches, and a larger soma and nucleus were seen on day 30. Round-shaped cells with scarce processes and enlarged body also referred to as the reactive-state or amoeboid form could be observed on day 60. Finally, OX42(+) and Iba1(+) small ovoid cells surrounded by 3 to 4 short and irregular nuclei appeared on day 120 post-BSSG administration suggesting cells in apoptosis (Figure 3). These changes suggest different activity states of microglia during chronic inflammation.

### 3.3. Time Course of Reactive Astrocyte Induction

**Figure 4:**
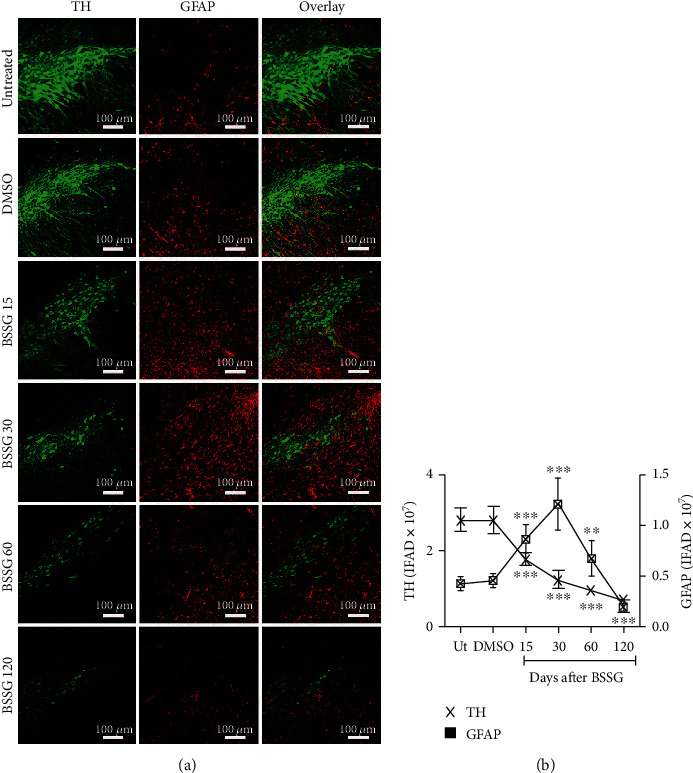
Time course of the astrocytic reactivity marker GFAP after a BSSG injection in the SNpc. (a) Representative micrographs of double immunofluorescence of TH and GFAP in untreated (Ut) control rats and rats at different times (shown at the left side of micrographs) after BSSG injection. (b) IFAD quantification for TH and GFAP during the experimental time course. The values are the mean ± SD from four anatomical levels. *n* = 3 independent rats in each time of each experimental condition. Two-way ANOVA and post hoc Bonferroni tests were applied for statistical analysis. The levels of significance were (^∗^) *P* < 0.05, (^∗∗^) *P* < 0.01, and (^∗∗∗^) *P* < 0.001 compared with the DMSO mock and Ut groups of the respective immunostaining.

**Figure 5:**
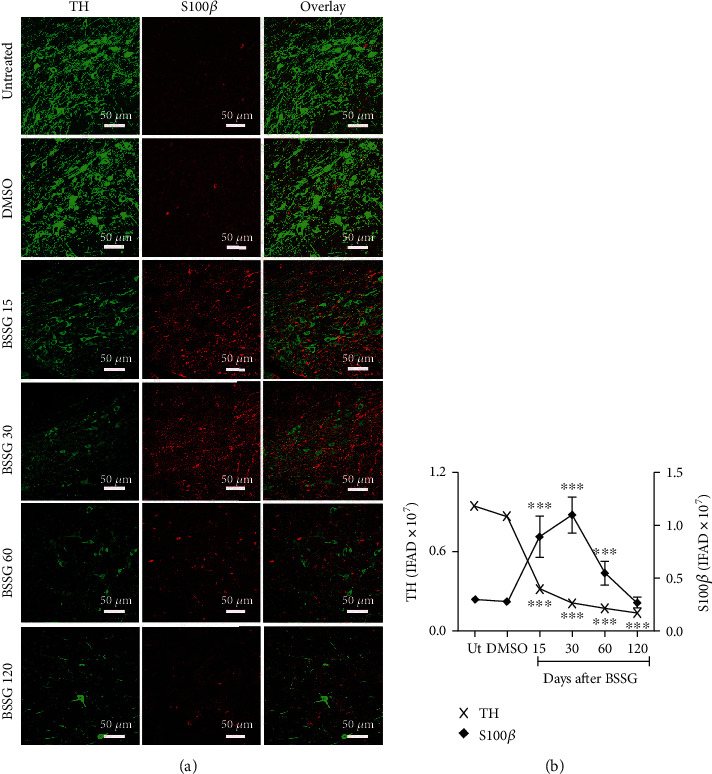
Time course of the reactive astrocyte induction marker S100*β* after a BSSG injection in the SNpc. (a) Representative micrographs of double immunofluorescence of TH and S100*β* in untreated (Ut) control rats and rats at different times (shown at the left side of micrographs) after BSSG injection. (b) IFAD quantification for TH and S100*β* during the experimental time course. The values are the mean ± SD from four anatomical levels. *n* = 3 independent rats in each time of each experimental condition. Two-way ANOVA and post hoc Bonferroni tests were applied for statistical analysis. (^∗∗∗^) indicates a *P* < 0.001 compared with the DMSO mock and Ut groups of the respective immunostaining.

**Figure 6:**
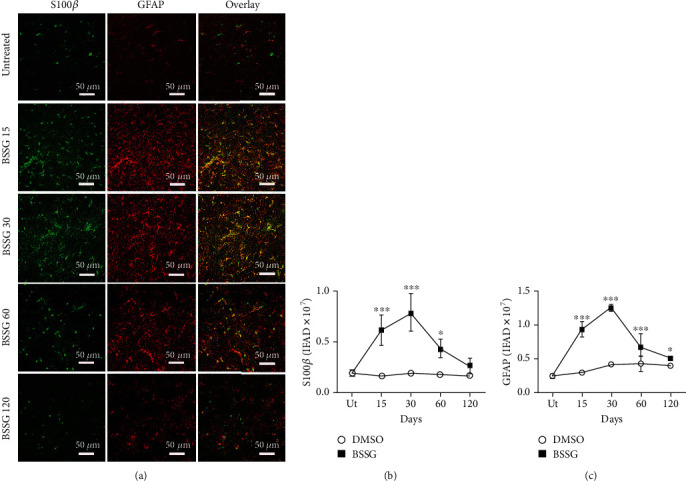
Colocalization of S100*β* and GFAP immunoreactivity after a BSSG injection in the SNpc. (a) Representative micrographs of the double immunofluorescence in untreated (Ut) control rats and rats at different times (shown at the left side of micrographs) after BSSG injection. IFAD quantification for S100*β* (b) and GFAP (c) during the experimental time course. The S100*β* and GFAP values for the mock rats correspond to the quantification in Supplementary Figure 5. The values are the mean ± SD from four anatomical levels. *n* = 3 independent rats in each time of each experimental condition. Two-way ANOVA and post hoc Bonferroni tests were applied for statistical analysis. The levels of significance were (^∗∗∗^) *P* < 0.001 and (^∗^) *P* < 0.05 compared with the DMSO mock and Ut groups of the respective immunostaining.

The astrocytic response and its association with dopaminergic neurodegeneration were assessed with GFAP intermediate filament [49] and S100*β* calcium-binding protein [12] immunoreactivity following BSSG administration. GFAP (Figure 4) and S100*β* (Figure 5) immunoreactivity increased in response to BSSG following the time course of microglial activation. The two astroglial markers were observed together with TH (+) neurons, which declined in number as expected (Figures 4 and 5). The astroglial markers were also assessed in parallel showing substantial colocalization, best observed during the time points of maximal microglial activation, on days 15 and 30 (Figure 6).

### 3.4. Presence of Neurotoxic A1 Reactive Astrocytes

**Figure 7:**
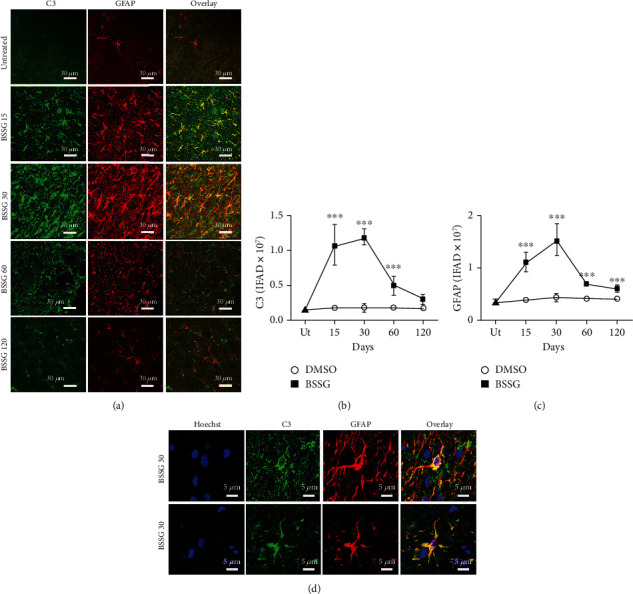
Colocalization of C3 and GFAP immunoreactivities after a BSSG injection in the SNpc. (a) Representative micrographs of the double in untreated (Ut) control rats and rats at different times (shown at the left side of micrographs) after BSSG injection. Immunofluorescence area density (IFAD) for C3 (b) and GFAP (c) was determined using the ImageJ software v.1.46r (National Institutes of Health, Bethesda, MD). The C3 and GFAP values for the mock rats correspond to the quantification in Supplementary Figure 6. (d) Details of cells coexpressing C3 and GFAP on day 30 after a BSSG injection in the SNpc. The values are the mean ± SD from four anatomical levels. *n* = 3 independent rats in each time of each experimental condition. Two-way ANOVA and post hoc Bonferroni tests were applied for statistical analysis. (^∗∗∗^) indicates a *P* < 0.001 compared with the DMSO mock and Ut groups of the respective immunostaining.

**Figure 8:**
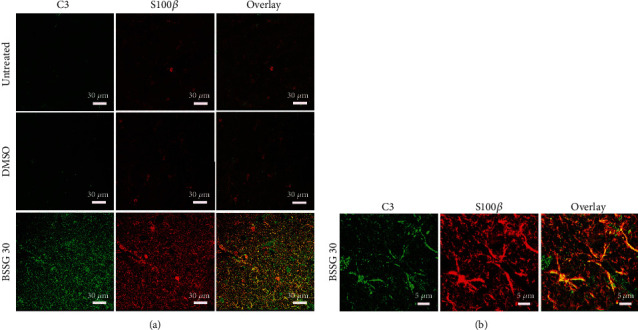
Cellular colocalization of C3 and S100*β* immunoreactivity on day 30 after a BSSG injection in the SNpc. (a) Representative micrographs of double immunofluorescence of C3 and S100*β* in untreated (Ut) control rats and rats injected with DMSO or BSSG. (b) Details of cells coexpressing C3 and S100*β* on day 30 after a BSSG injection in the SNpc.

To identify the presence of neurotoxic A1 reactive astrocytes, the specific marker C3, characteristic for these astrocytes [10], was monitored together with GFAP or S100*β* in double immunofluorescence assays. C3-immunoreactive cells were absent in the SNpc of untreated and mock groups (Figures 7 and 8). In contrast, a significant number of C3(+) cells appeared on day 15 after BSSG administration, and the cell amount significantly augmented on day 30 to decrease afterward. The pattern of appearance over time for GFAP(+) cells (Figures 7(a) and 7(c)) and S100*β*(+) cells (Figure 8) was consistent with reactive astrocytic induction on days 15 and 30 post-BSSG administration. C3(+) cells colocalized with GFAP(+) cells and S100*β*(+) cells on day 30 after BSSG administration, suggesting the presence of A1 cytotoxic astrocytes. Closer views of the colocalization between the three markers showed significant overlap but also an independent expression of these proteins in some cells (Figures 7(d) and 8(b)).

### 3.5. Leukocyte Infiltration

**Figure 9:**
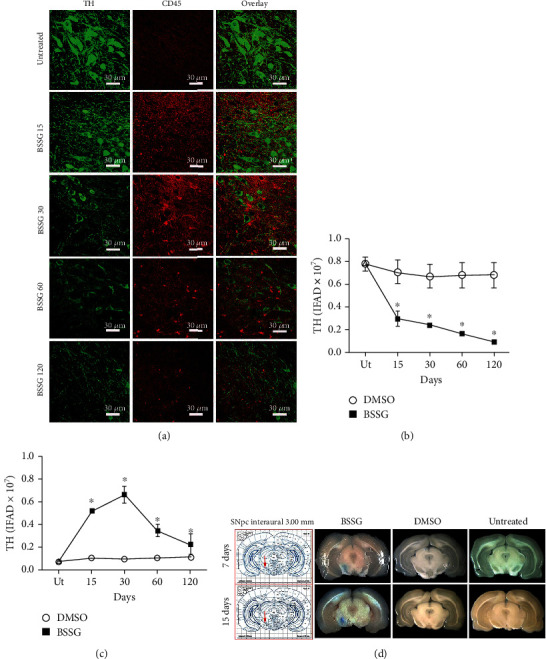
Time course of leukocyte infiltration after a BSSG injection in the SNpc. (a) Representative micrographs of double immunofluorescence of TH and CD45 in untreated (Ut) control rats and rats at different times (shown at the left side of micrographs) after BSSG injection. IFAD quantification for TH and CD45 during the experimental time course. The (b) TH and (c) CD45 values for the mock rats correspond to the quantification in Supplementary Figure 7. The values are the mean ± SD from four anatomical levels. *n* = 3 independent rats in each time of each experimental condition. Two-way ANOVA and post hoc Bonferroni tests were applied for statistical analysis. (^∗^) indicates a *P* < 0.05 compared with the DMSO mock and Ut groups of the respective immunostaining. (d) Representative photographs of brain slices after intravenous injection of the Evans blue dye on the days shown at the left of each row.

In neuroinflammation, activated microglia release proinflammatory cytokines, which promote the BBB opening, allowing leukocytes to pass from circulation to the brain parenchyma. Accordingly, CD45 immunoreactive leukocytes [50], which are absent in the SNpc of untreated and DMSO mock groups (Figures 9(a) and 9(c)), appeared on day 15 after BSSG administration and then followed the time course of microglial activation. Once again, the TH (+) cells followed the pattern described in the previous assays (Figures 9(a) and 9(b)). The presence of Evans blue dye in the SNpc confirmed the loss of BBB integrity on days 7 and 15 following BSSG administration (Figure 9(d)). These results suggest that the BSSG treatment resulted in the infiltration of leukocytes across a leaky BBB, as a consequence of neuroinflammation.

### 3.6. Proinflammatory Cytokines

**Figure 10:**
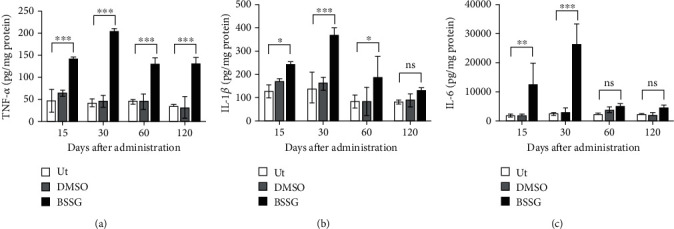
Levels of proinflammatory cytokines in the *substantia nigra* after BSSG intranigral injection. ELISA was used to measure protein levels of (a) TNF-*α*, (b) IL-1*β*, and (c) IL-6. All values represent the mean ± SD (*n* = 3 rats per time point per experimental condition). Two-way ANOVA and Bonferroni post hoc test were applied for statistical analysis. The levels of significance were ^∗^*P* < 0.05, ^∗∗^*P* < 0.01, and ^∗∗∗^*P* < 0.001. ns: not significant.

TNF-*α*, IL-1*β*, and IL-6 were evaluated in the *substantia nigra* through ELISA (Figure 10). The basal levels expressed in pg/mg of protein were 41.5 ± 12.8 for TNF-*α*, 127.4 ± 31.7 for IL-1*β*, and 1619.9 ± 761.6 for IL-6.The DMSO vehicle injection did not significantly change the basal levels for the three proinflammatory cytokines (Figure 10). In contrast, the levels of those proinflammatory cytokines were significantly and differentially increased by the BSSG administration as compared with the Ut and DMSO groups. All proinflammatory cytokine levels were markedly higher from day 15 to day 30, corresponding to the period of BBB opening. TNF-*α* levels remained high throughout the time points included in this study, whereas IL-1*β* was induced until day 60, and IL-6 was not detected beyond day 30 (Figure 10). These results are consistent with the conclusion that BSSG injection caused chronic neuroinflammation.

## 4. Discussion

This report provides evidence that A1 neurotoxic reactive astrocytes contribute to chronic neuroinflammation elicited by a single intranigral administration of BSSG. Activated microglia may be involved in the induction of A1 astrocytes from GFAP(+) and S100*β*(+) cell populations through the release of proinflammatory cytokines [10, 18], which also could be released by infiltrating immune cells, neurons, and possibly by BBB-endothelial cells known to express IL-1*β* and IL-6 genes [40, 41]. *In vitro*, conditioned medium from LPS-activated microglia induces A1 reactive astrocytes, which rapidly kill neurons by secreting unidentified neurotoxins [10]. Based on this evidence, we propose that A1 reactive astrocytes could also participate in the death of dopaminergic neurons in the BSSG-treated animals, as shown here and also in the oral administration model [34]. This suggestion is further supported by the identification of A1 astrocytes in postmortem samples of PD brains [10].

Reactive oxygen and nitrogen species (RONS) are notable contributors to neuronal death in neuroinflammation through the irreversible oxidative or nitrosative injury to biomolecules [44, 51]. Here, we found that BSSG induced a fast NO production that remained increased during the first month after its intranigral administration. This fast increase in NO production might be due to the contribution of neuronal nitric oxide synthase (nNOS) in dopaminergic neurons that is stimulated by NMDA receptor-mediated excitotoxicity [52, 53]. This pathological process seems to mediate the BSSG-triggered dopaminergic loss [32] because these neurons express NMDA receptors [54, 55] and are sensitive to glutamate [56]. The increase in glutamate can be caused by pathological *α*-synuclein aggregates [57] known to occur in the acute [38] and chronic [35] BSSG administration. The sustained NO production that coincided with the periods of microglia activation, reactive astrocyte induction, and leukocyte infiltration can be due to the iNOS expression in those inflammatory cells [58, 59]. Nitrosative/oxidative stress affects particularly dopaminergic neurons because they lack an efficient antioxidant defense showing low levels of glutathione and moderate catalase, superoxide dismutase, and glutathione peroxidase activities [60, 61]. Besides, the SNpc is a nucleus with a high microglial population, which can detect a minimum imbalance of oxidative stress and mount a fast response [62, 63] that is potentiated by the infiltrating immune cells. We also found that BSSG-induced nitrosative stress coincided with the periods of reactive astrocyte induction and reduction of dopaminergic neuron viability. Since S100*β* can stimulate NO production [12, 13], then S100*β* released by reactive astrocytes after BSSG intranigral administration may also contribute to nitrosative stress and consequently, to the death of dopaminergic neurons. Since S100*β*(+) cells colocalize with C3(+) cells, NO could be one of the unknown neurotoxins that mediate the harmful effect of A1 reactive astrocytes on dopaminergic neurons [14]. Interestingly, oxidative stress did not occur until day 120 after the BSSG administration, when the maximum decrease in dopaminergic neuron population was attained. This result suggests that apoptosis of dopaminergic neurons occurs during the NO production period and that other cells undergo lipid peroxidation in the late phase of neuroinflammation.

Microglia are parenchymal CNS macrophages that perform a surveillance function, scanning the entire brain tissue in the resting state [64]. Upon detecting a hazardous stimulus, they become rapidly activated, changing shape and acquiring immunological functions [44, 65]. In the BSSG injection model, microglia activation evidenced through OX42 and Iba1 markers and cell morphology [44, 48] developed progressively up to day 30 after the injection. In contrast, shape changes appeared for more prolonged times as compared with laser stimulation (up to 5.5 hours) [64], traumatic brain injury [66], neurotoxic lesions (24 hours after injury) [43], and LPS stimulation (up to 168 hours) [44] models. The relatively long duration of the response observed suggests that microglia was not activated by the direct hazardous stimulus of BSSG but by downstream effects. A possible activation stimulus could be the pathological *α*-synuclein aggregates that appear as a delayed outcome of stereotaxic [37] and oral [34] BSSG administration models. Accumulating evidence supports the notion that *α*-synuclein aggregates activate microglial cells by different mechanisms, including nitrosative/oxidative stress [19–22] and immune cell infiltration [23, 24, 44]. Here, these two events were associated with cell morphology and the increased OX42 and Iba1 markers over time, suggesting their participation in the development of microglia activation. This putative activation was also associated with the time course of proinflammatory cytokines (TNF-*α*, IL-1*β*, and IL-6), which were present throughout the study (120 days). Such interrelationship supports the microglia source of those cytokines, although they could also come from the infiltrating immune cells that showed a similar time course. Together, these findings show that the BSSG-induced neuroinflammation is chronic, similar to the one occurring in Parkinson's disease but in contrast to the acute response induced by LPS [44], traumatic brain injury [66], or transient ischemia [67]. In the acute neuroinflammation by LPS, TNF-*α* and IL-1*β* reached maximum levels at 5 h after the lesion and at 8 h for IL-6 [44]. The balance between pro- and anti-inflammatory cytokinesis is crucial for controlling the neuroinflammatory response, as recently shown in the acute LPS-induced neuroinflammation in the *substantia nigra* [44]. However, this issue was not explored in SNpc injected because the profound dopaminergic neurodegeneration suggests that the contribution of anti-inflammatory cytokines might be smaller than that of proinflammatory cytokines. Similar to A2 reactive astrocytes, it would be relevant to study the balance between pro- and anti-inflammatory cytokinesis to gain insight into the control of the immune response in those brain regions where neuroinflammation is emerging by the presence of pathological *α*-synuclein [37, 38].

Following traumatic brain injury, A1 neurotoxic astrocytes appeared before microglia, suggesting the existence of microglia-independent induction mechanisms *in vivo* [66]. In contrast, after BSSG injection, A1 neurotoxic astrocytes followed a similar time course with activated microglia and the increase in proinflammatory cytokine levels. These results do not identify the primary event for A1 reactive astrocyte induction because an earlier time after the BSSG administration was not explored. Nevertheless, it is possible that in chronic inflammation, a more complex interaction between astrocytes and microglia exists. For instance, A1 astrocytes induced in a microglia-independent manner might be themselves a cause of microglial activation [42, 68] and microglia migration by secreting S100*β* protein [69]. The colocalization of C3 and S100*β* immunoreactivities observed in our study would support such a notion. The BSSG stereotaxic model can help clarify the mechanism of A1 astrocyte induction in chronic neuroinflammation. Contrary to neurotoxic A1 reactive astrocytes, A2 reactive astrocytes that are identified by the specific marker S100A10 have been postulated to be protective based on transcriptome analysis showing upregulation of many neurotrophic factors and thrombospondins, which promote synapse repair in animal models of acute injury [10, 70]. In the stereotaxic BSSG model, the severe and progressive dopaminergic neurodegeneration in the *substantia nigra* suggests that A2 reactive astrocytes do not play a significant protective role. The intranigral BSSG model is known to trigger pathological *α*-synuclein aggregates that spread from the neurotoxin application site to diverse brain regions, possibly producing neuroinflammation, as suggested by behavioral impairments [37, 38]. Then, it would be relevant to study the induction of A1 and A2 reactive astrocytes and determine the balance between cells with phenotype A1 and A2 in those brain regions where neuroinflammation is emerging.

A failure of BBB permeability in Parkinson's disease [25] permits the infiltration of lymphocytes [23, 24] that could generate an autoimmune response to *α*-synuclein aggregates [29]. In the stereotaxic model, BBB opening is of such magnitude as shown by the Evans blue assay that it enabled a great infiltration of bone marrow- (BM-) derived cells that can be detected by the antibody to CD45, which is a pan-leukocyte marker [71]. Therefore, it is plausible to assume that macrophages, lymphocytes, CD4, CD8, TReg, and NK infiltrate the *substantia nigra*. The immune response of such diverse immune cells does potentiate the response of resident defensive cells, thus contributing to the severity and irreversibility of BSSG-induced dopaminergic neurodegeneration, as shown here and by other works [34, 35, 37, 38]. Furthermore, the increased BBB permeability may also enable the invasion to the brain of microbiota metabolic products, peripheral *α*-synuclein aggregates, and mediators of the innate immune system resulting from gut dysbiosis and/or bacterial overgrowth, which are implicated in the brain-gut-microbiota axis in Parkinson's disease [72–74]. A contrary flow from the brain to blood circulation and peripheral organs of harmful cell decomposition products, proinflammatory cytokines, and pathological *α*-synuclein aggregates triggered by the BSSG-induced severe neuroinflammation might also occur [74, 75]. The BSSG stereotaxic model represents a valuable tool to clarify this hypothesis and advance the knowledge in the pathogenesis of Parkinson's disease.

In this work, DMSO was used to dissolve BSSG because pyridine used to solubilize BSSG [76] caused necrosis in the *substantia nigra* (data not shown). Although concentrated DMSO may be a methodological drawback, its single administration (1 *μ*L) did not significantly change any variables studied as compared with the untreated healthy animals. These results agree with a recent report showing that DMSO does not trigger apoptosis or senescence in the *substantia nigra* cells, neither elicits changes in the cytoskeleton or density of dendritic spines [38] or behavioral alterations [37]. Other authors that used 100% DMSO also do not report damage or altered function *in vivo* [77–80]. In contrast, other studies have reported that DMSO is toxic in cell cultures. For instance, it affects cell proliferation and production of proinflammatory cytokines in cultures of peripheral blood lymphocytes [81] and impairs mitochondrial integrity and membrane potential in cultured astrocytes [81, 82]. However, our results with lipoperoxidation assay suggest that DMSO did not damage cell membranes at times here evaluated, neither augmented proinflammatory cytokines. A possible explanation is that the glymphatic system [83] diluted the DMSO concentration in the one *μ*L injected, thus dampening its toxicity. On the contrary, cultured cells are directly exposed to DMSO because they lack the defensive physiological mechanisms present *in vivo*. It would be interesting to evaluate whether DMSO triggers neuroinflammation signaling in other cells, such as oligodendrocytes and ependymal cells in the stereotaxic BSSG model.

The chronic oral administration of BSSG to Sprague-Dawley rats faithfully models Parkinson's disease, reproducing the development of motor and nonmotor behavior impairments and insoluble *α*-synuclein appearance according to the Braak stages of PD [34]. Likewise, intranigral administration of BSSG replicates similar characteristics, such as the progression of behavioral alterations, dopaminergic neuron loss, and the presence of Lewy body-like synuclein aggregations in a shorter time [37]. The hallmark of the aggregates triggered by an acute BSSG intranigral injection is the ability to spread in a prion-like manner to anatomically interconnected and noninterconnected regions in the whole brain [38]. Furthermore, behavioral tests have shown that motor and nonmotor impairments could result from neurological damage in those diverse regions [37, 38]. These antecedents and the finding that pathological *α*-synuclein aggregates can induce neuroinflammation [84, 85] strongly suggest that the acute BSSG intranigral administration also leads to neuroinflammation in the whole brain. Thus, systematic or local BSSG administration models can be used to clarify the mechanisms of chronic neuroinflammation and validate the emerging therapeutic approaches for Parkinson's disease, such as anti-inflammatory gene therapy [45], anti-*α*-synuclein immunotherapy [86, 87], and A1 reactive astrocyte induction blocking therapy [17].

## 5. Conclusion

Our data show that a single intranigral BSSG injection triggers chronic neuroinflammation in the SNpc and degeneration of dopaminergic neurons. All markers of neuroinflammation, including those for neurotoxic A1 reactive astrocytes, showed similar changes over time with a maximum elevation in the first month, whereas the loss of dopaminergic neurons was progressive to reach a drastic decline on day 120 postadministration. These data suggest that neuroinflammation triggers dopaminergic neurodegeneration via neurotoxic A1 reactive astrocytes. However, infiltrating BM-derived cells in the SNpc due to BBB breakdown may also participate in the neuronal loss via an autoimmune response against *α*-synuclein aggregates present in the SNpc of both BSSG administration models [34, 37]. Besides, the sustained high levels of proinflammatory cytokines resulting from activated microglial cells, reactive astrocytes, infiltrating BM-derived cells [10, 39, 65], and possibly BBB-endothelial cells [40, 41] could account for the severity of the BSSG-induced neuroinflammation in the SNpc. Further studies are needed to explore control mechanisms of neuroinflammation, such as the role of A2 reactive astrocytes and anti-inflammatory cytokines. The BSSG stereotaxic administration in the rat is an easy model of Parkinson's disease that will help to answer open questions on mechanisms of chronic neuroinflammation and neurodegeneration. Also, emerging therapies for Parkinson's disease can be validated in this rat model of chronic neuroinflammation.

## Data Availability

The data that support the findings of this study are available from the corresponding author upon reasonable request.

## Supplementary Materials

Supplementary Materials Supplementary Figure 1: illustration of experimental design. (a) Timeline of the tests performed after the injection of BSSG or DMSO. Ut: untreated group. (b) Table with the number of animals used per assays every time point and group evaluated. Supplementary Figure 2: representative micrographs of TH and OX42 double immunofluorescence in untreated control and mock rats at different times (shown on the left side of micrographs) after DMSO injection. The immunofluorescence area (IFAD) density was measured in four anatomical levels per rat with the ImageJ software v.1.46r 40 (National Institutes of Health; Bethesda, MD). The mean ± SD values are shown in the graph of Figure 2(b) for TH and Figure 2(c) for OX42. *n* = 3 independent rats in each time of each experimental condition. Supplementary Figure 3: a single intranigral BSSG administration increases the Iba1(+) cell population over time. (a) Representative micrographs of Iba1 immunohistochemistry in the SNpc of a rat per condition (shown at the left side micrographs). (b) The graph shows the area density that was determined with the ImageJ software v.1.46r (National Institutes of Health; Bethesda, USA). The mean ± SD (*n* = 3 slices in each time of each experimental condition). One-way ANOVA and Newman-Keuls post hoc tests were applied for statistical analysis. (^∗∗∗^) *P* < 0.001, (^∗^) *P* < 0.05 as compared with the values of DMSO mock and untreated groups. Supplementary Figure 4: a single intranigral BSSG administration increases the Iba1(+) cell population over time. (a) Representative micrographs of Iba1 immunofluorescence in the SNpc of a rat per condition (shown at the left side micrographs). (b) The graph shows the immunofluorescence area density (IFAD) that was determined with the ImageJ software v.1.46r (National Institutes of Health; Bethesda, USA). The mean ± SD (*n* = 3 slices in each time of each experimental condition). One-way ANOVA and Newman-Keuls post hoc tests were applied for statistical analysis. (^∗∗∗^) *P* < 0.001, (^∗^) *P* < 0.05 as compared with the values of DMSO mock and untreated groups. Supplementary Figure 5: representative micrographs of S100*β* and GFAP double immunofluorescence in the SNpc of untreated control rats and mock rats at different times (shown at the left side of micrographs) after DMSO injection. The immunofluorescence area density (IFAD) was measured in four anatomical levels per rat with the ImageJ software v.1.46r 40 (National Institutes of Health; Bethesda, MD). The mean ± SD values are shown in the graph of Figure 6(b) for S100*β* and Figure 6(c) for GFAP. *n* = 3 independent rats in each time of each experimental condition. Supplementary Figure 6: representative micrographs of the double immunofluorescence for C3 and GFAP in the SNpc of untreated control rats and mock rats at different times (shown at the left side of micrographs) after DMSO injection. The immunofluorescence area density (IFAD) was measured in four anatomical levels per rat with the ImageJ software v.1.46r 40 (National Institutes of Health; Bethesda, MD). The mean ± SD values are shown in the graph of Figure 7(b) for C3 and Figure 7(c) for GFAP. *n* = 3 independent rats in each time of each experimental condition. Supplementary Figure 7: representative micrographs of the double immunofluorescence for TH and CD45 in untreated control rats and mock rats at different times (shown at the left side of micrographs) after DMSO injection. The immunofluorescence area density (IFAD) was measured in four anatomical levels per rat with the ImageJ software v.1.46r 40 (National Institutes of Health; Bethesda, MD). The mean ± SD values are shown in the graph of Figure 9(b) for TH and Figure 9(c) for CD45. *n* = 3 independent rats in each time of each experimental condition. Supplementary Figure 8: merged image sets below display double immunofluorescence assays in three independent rats per time point after DMSO or BSSG intranigral administration in comparison with untreated rats. Headings indicate the double immunostaining type. Left labels show the time and experimental condition. Legends indicate the main figure number where the quantification data appear. Set 1: DMSO effect on dopaminergic neurons (TH) and microglia (OX42) compared with untreated controls. Quantification data appear in the graph of Figure 2(b) for TH and Figure 2(c) for OX42. Set 2: DMSO effect on dopaminergic neurons (TH) and astrocytes (GFAP) compared with untreated controls. Quantification data appear in the graph of Figure 4(b) for TH and Figure 4(c) for GFAP. Set 3: DMSO effect on dopaminergic neurons (TH) and astrocytes (S100*β*) compared with untreated controls. Quantification data appear in the graph of Figure 5(b) for TH and Figure 5(b) for S100*β*. Set 4: DMSO effect on astrocytes (S100*β*) and (GFAP) compared with untreated controls. Quantification data appear in the graph of Figure 6(b) for S100*β* and Figure 6(c) for GFAP. Set 5: DMSO effect on astrocytes (C3) and (GFAP) compared with untreated controls. Quantification data appear in the graph of Figure 7(b) for C3 and Figure 7(c) for GFAP. Set 6: DMSO effect on dopaminergic neurons (TH) and leukocytes (CD45) compared with untreated controls. Quantification data appear in the graph of Figure 9(b) for TH and Figure 9(c) for CD45. Set 7: BSSG effect on dopaminergic neurons (TH) and microglia (OX42) compared with untreated controls. Quantification data appear in the graph of Figure 2(b) for TH and Figure 2(c) for OX42. Set 8: BSSG effect on dopaminergic neurons (TH) and astrocytes (GFAP) compared with untreated controls. Quantification data appear in the graph of Figure 4(b) for TH and Figure 4(b) for GFAP. Set 9: BSSG effect on dopaminergic neurons (TH) and astrocytes (S100*β*) compared with untreated controls. Quantification data appear in the graph of Figure 5(b) for TH and Figure 5(b) for S100*β*. Set 10: BSSG effect on astrocytes (S100*β*) and (GFAP) compared with untreated controls. Quantification data appear in the graph of Figure 6(b) for S100*β* and Figure 6(c) for GFAP. Set 11: BSSG effect on astrocytes (C3) and (GFAP) compared with untreated controls. Quantification data appear in the graph of Figure 7(b) for C3 and Figure 7(c) for GFAP. Set 12: BSSG effect on dopaminergic neurons (TH) and leukocytes (CD45) compared with untreated controls. Quantification data appear in the graph of Figure 9(b) for TH and Figure 9(c) for CD45.
