# Supplementary material for: Intranigral Administration of β-Sitosterol-β-D-Glucoside Elicits Neurotoxic A1 Astrocyte Reactivity and Chronic Neuroinflammation in the Rat Substantia Nigra
Source: J Immunol Res. 2020 Nov 16;2020:5907591. doi: 10.1155/2020/5907591 (PMC7685831; doi:10.1155/2020/5907591)
Supplement: Supplementary Materials — Supplementary Figure 1: illustration of experimental design. (a) Timeline of the tests performed after the injection of BSSG or DMSO. Ut: untreated group. (b) Table with the number of animals used per assays every time point and group evaluated. Supplementary Figure 2: representative micrographs of TH and OX42 double immunofluorescence in untreated control and mock rats at different times (shown on the left side of micrographs) after DMSO injection. The immunofluorescence area (IFAD) density was measured in four anatomical levels per rat with the ImageJ software v.1.46r 40 (National Institutes of Health; Bethesda, MD). The mean ± SD values are shown in the graph of Figure 2(b) for TH and Figure 2(c) for OX42. n = 3 independent rats in each time of each experimental condition. Supplementary Figure 3: a single intranigral BSSG administration increases the Iba1(+) cell population over time. (a) Representative micrographs of Iba1 immunohistochemistry in the SNpc of a rat per condition (shown at the left side micrographs). (b) The graph shows the area density that was determined with the ImageJ software v.1.46r (National Institutes of Health; Bethesda, USA). The mean ± SD (n = 3 slices in each time of each experimental condition). One-way ANOVA and Newman-Keuls post hoc tests were applied for statistical analysis. (∗∗∗) P < 0.001, (∗) P < 0.05 as compared with the values of DMSO mock and untreated groups. Supplementary Figure 4: a single intranigral BSSG administration increases the Iba1(+) cell population over time. (a) Representative micrographs of Iba1 immunofluorescence in the SNpc of a rat per condition (shown at the left side micrographs). (b) The graph shows the immunofluorescence area density (IFAD) that was determined with the ImageJ software v.1.46r (National Institutes of Health; Bethesda, USA). The mean ± SD (n = 3 slices in each time of each experimental condition). One-way ANOVA and Newman-Keuls post hoc tests were applied for statistical analysis. (∗∗∗) P [file 5907591.f1.pdf]

Supplementary Material

(a)

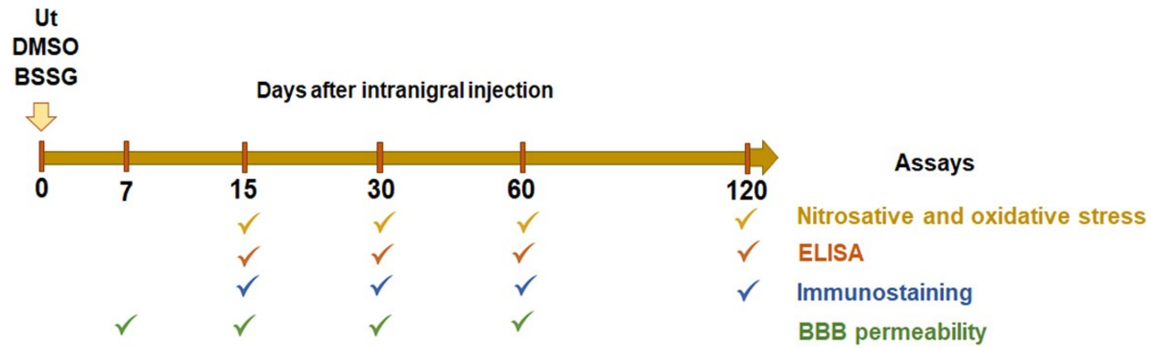

(b)

Number of animals

Per experiment		Per times (X 4)	Per group
• Nitrosative and oxidative stress	*n* = 4	*n* = 16	
• ELISA	*n* = 3	*n* = 12	
• BBB permeability	*n* = 3	*n* = 12	BSSG *n* = 40 + 12 = 52
		**Subtotal = 40**	DMSO *n* = 40 + 12 = 52
			Ut *n* = 40 + 3 = 43
		n = 12 BSSG	
• Immunostainig	*n* = 3	*n* = 12 DMSO	
		n = 3 (x1) Ut	**Total = 147**

Supplementary Figure 1: Illustration of experimental design. (a) Timeline of the tests performed after the injection of BSSG or DMSO. Ut = untreated group. (b) Table with the number of animals used per assays every time point and group evaluated.

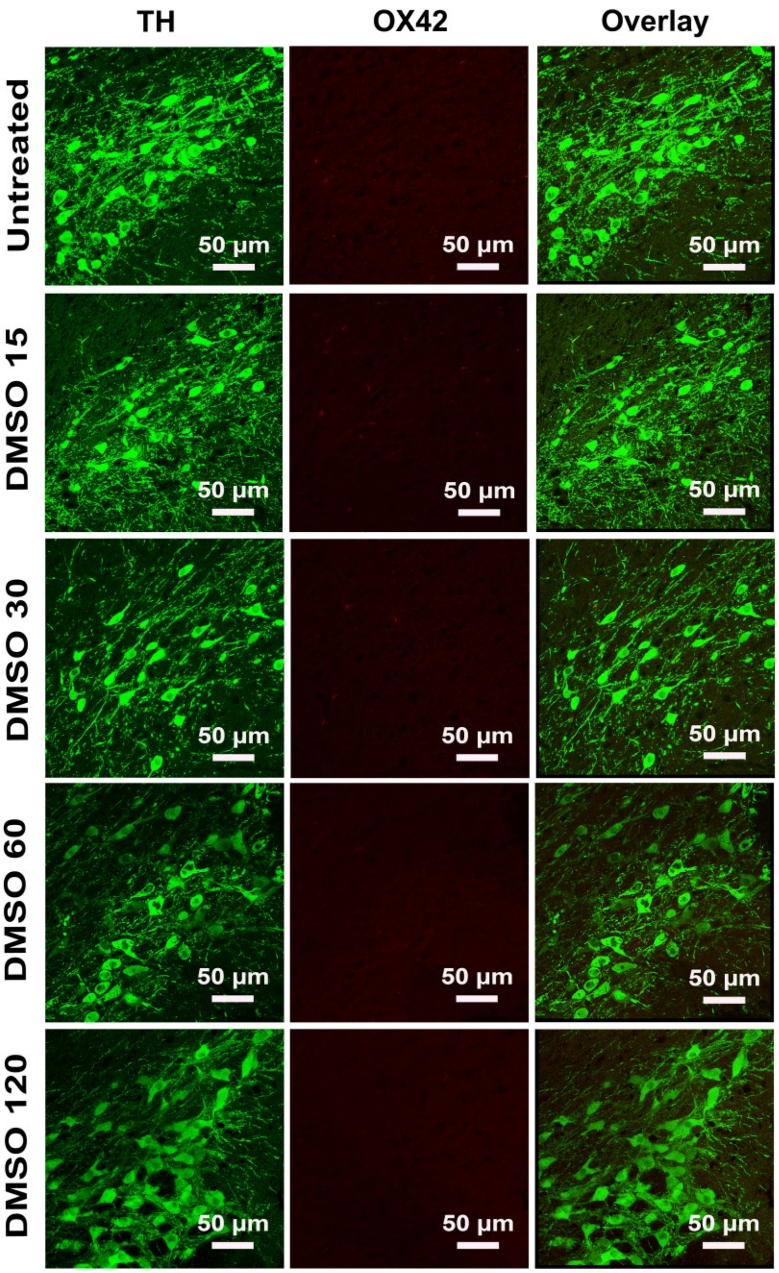

Supplementary Figure 2: Representative micrographs of TH and OX42 double immunofluorescence in untreated control and mock rats at different times (shown on the left side of micrographs) after DMSO injection. The immunofluorescence area (IFAD) density was measured in four anatomical levels per rat with ImageJ software v.1.46r 40 (National Institutes of Health; Bethesda, MD). The mean ± SD values are shown in the graph of Figure 2(b) for TH and Figure 2(c) for OX42. *n* = 3 independent rats in each time of each experimental condition.

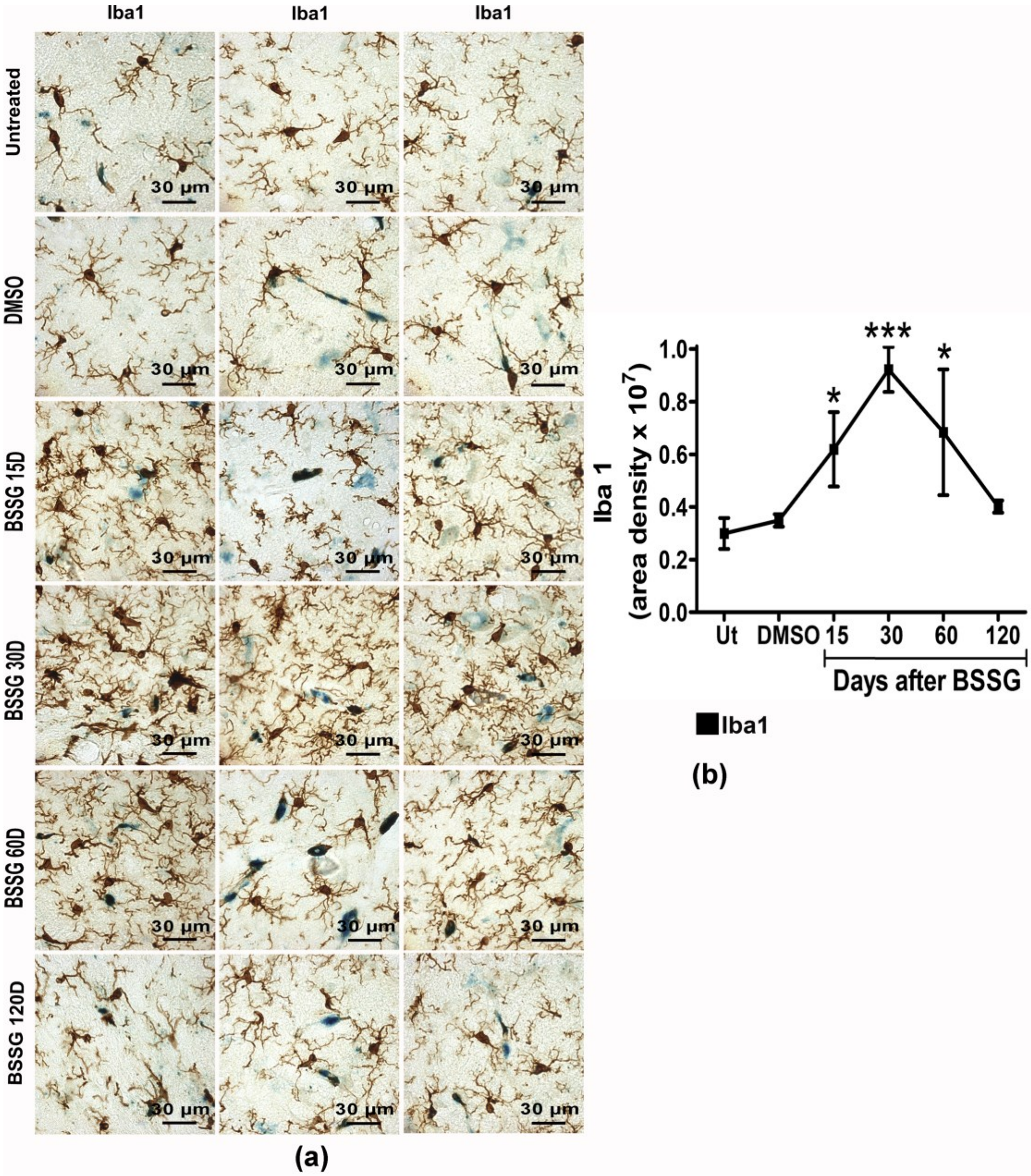

Supplementary Figure 3: A single intranigral BSSG administration increases the Iba1(+) cell population over time. a) Representative micrographs of Iba1 immunohistochemistry in the SNpc of a rat per condition (shown at the left side micrographs). b) The graph shows the area density that was determined with the ImageJ software v.1.46r (National Institutes of Health; Bethesda, USA). The mean ± SD (*n* = 3 slices in each time of each experimental condition). One-way ANOVA and Newman-Keuls post hoc tests were applied for statistical analysis. (***) $P < 0.001$, (*) $P < 0.05$ as compared with the values of DMSO mock and untreated groups.

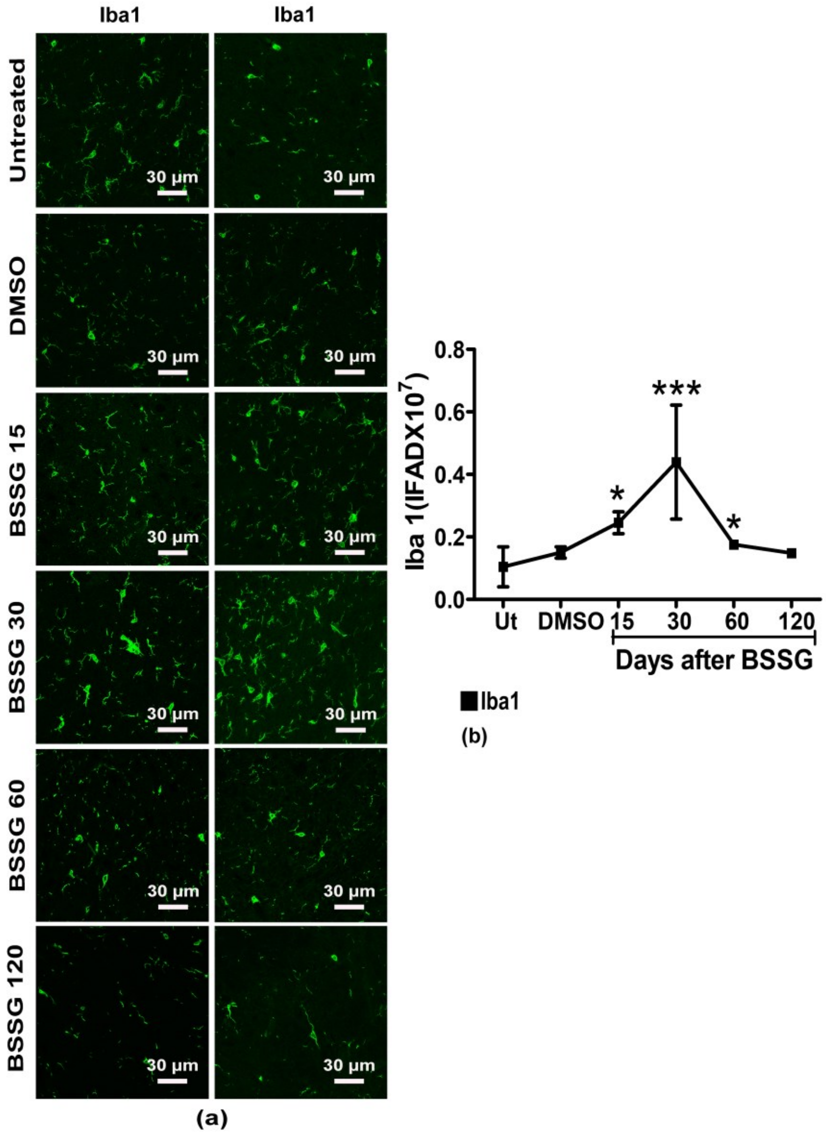

Supplementary Figure 4: A single intranigral BSSG administration increases the Iba1(+) cell population over time. a) Representative micrographs of Iba1 immunofluorescence in the SNpc of a rat per condition (shown at the left side micrographs). b) The graph shows the immunofluorescence area density (IFAD) that was determined with the ImageJ software v.1.46r (National Institutes of Health; Bethesda, USA). The mean ± SD (n = 3 slices in each time of each experimental condition). One-way ANOVA and Newman-Keuls post hoc tests were applied for statistical analysis. (***) $P < 0.001$, (*) $P < 0.05$ as compared with the values of DMSO mock and untreated groups.

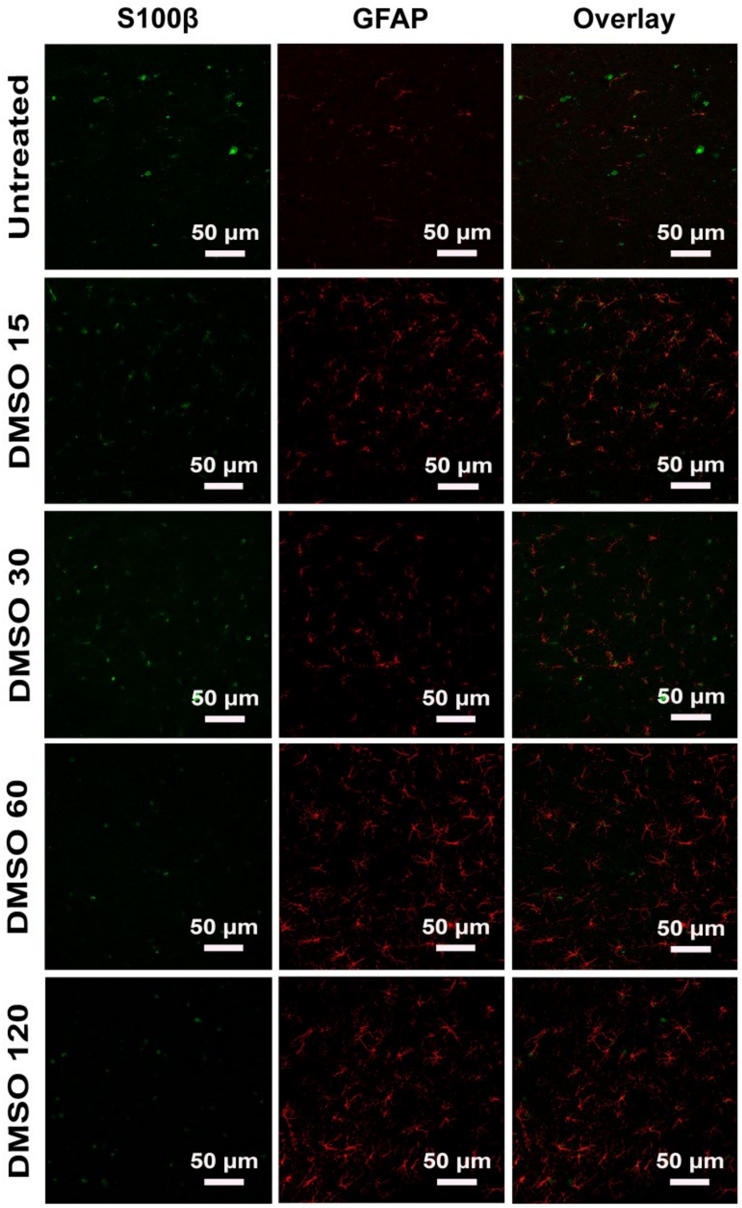

Supplementary Figure 5: Representative micrographs of S100β and GFAP double immunofluorescence in the SNpc of untreated control rats and mock rats at different times (shown at the left side of micrographs) after DMSO injection. The immunofluorescence area density (IFAD) was measured in four anatomical levels per rat with ImageJ software v.1.46r 40 (National Institutes of Health; Bethesda, MD). The mean ± SD values are shown in the graph of Figure 6(b) for S100β and Figure 6(c) for GFAP. *n* = 3 independent rats in each time of each experimental condition.

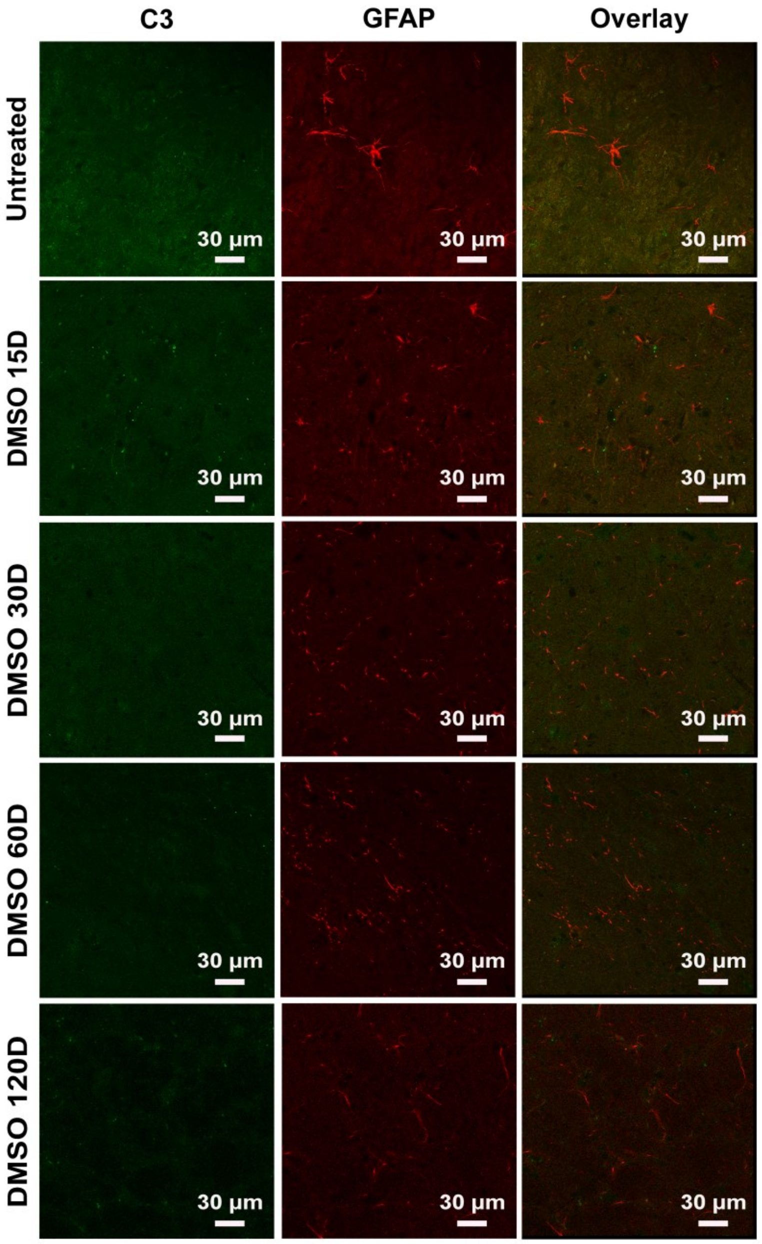

Supplementary Figure 6: Representative micrographs of the double immunofluorescence for C3 and GFAP in the SNpc of untreated control rats and mock rats at different times (shown at the left side of micrographs) after DMSO injection. The immunofluorescence area density (IFAD) was measured in four anatomical levels per rat with ImageJ software v.1.46r 40 (National Institutes of Health; Bethesda, MD). The mean ± SD values ae shown in the graph of Figure 7(b) for C3 and Figure 7(c) for GFAP. *n* = 3 independent rats in each time of each experimental condition.

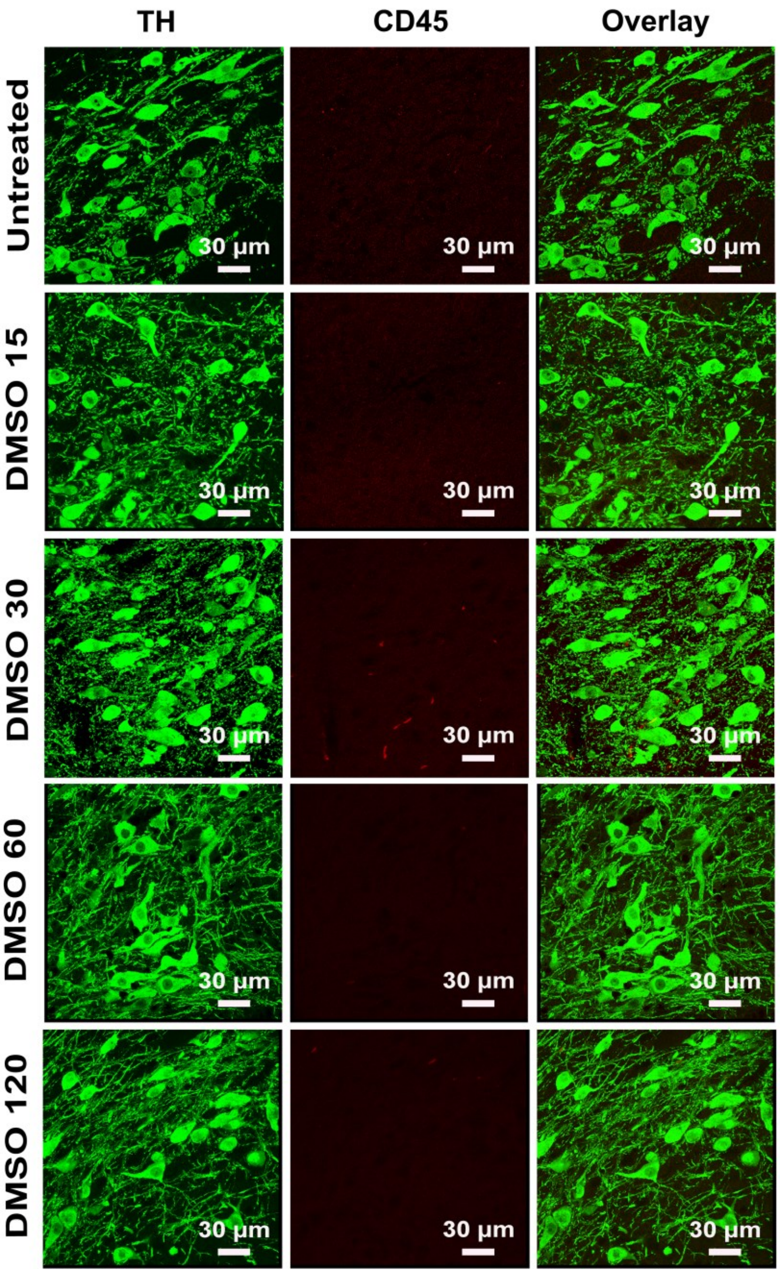

Supplementary Figure 7: Representative micrographs of the double immunofluorescence for TH and CD45 in untreated control rats and mock rats at different times (shown at the left side of micrographs) after DMSO injection. The immunofluorescence area density (IFAD) was measured in four anatomical levels per rat with ImageJ software v.1.46r 40 (National Institutes of Health; Bethesda, MD). The mean ± SD values are shown in the graph of Figure 9(b) for TH and Figure 9(c) for CD45. *n* = 3 independent rats in each time of each experimental condition.

Supplementary Figure 8: Merged image sets below display double immunofluorescence assays in three independent rats per time point after DMSO or BSSG intranigral administration in comparison with untreated rats. Headings indicate the double immunostaining type. Left labels show the time and experimental condition. Legends indicate the main figure number where the quantification data appear.

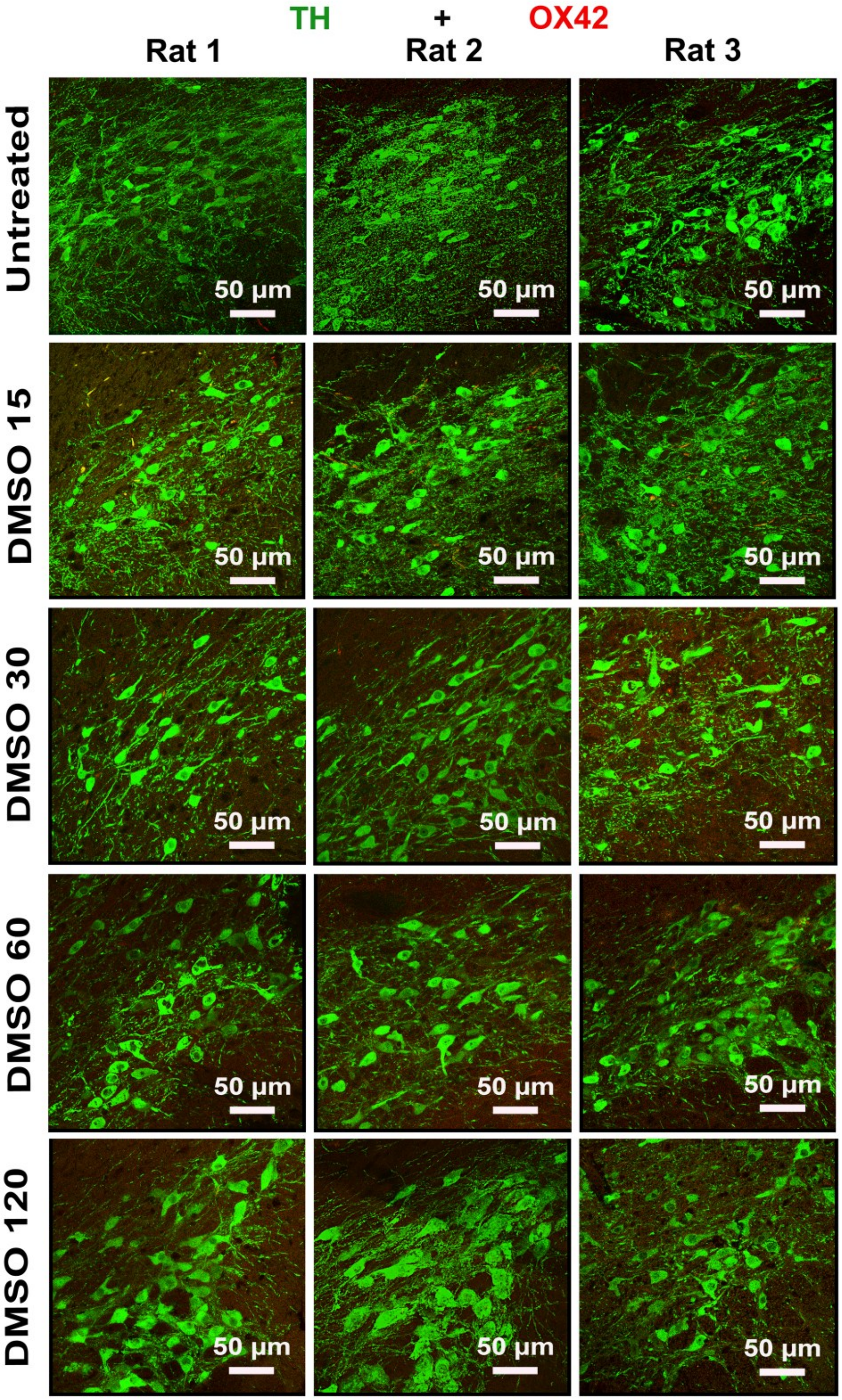

Set 1: DMSO effect on dopaminergic neurons (TH) and microglia (OX42) compared with untreated controls. Quantification data appear in the graph of Figure 2(b) for TH and Figure 2(c) for OX42.

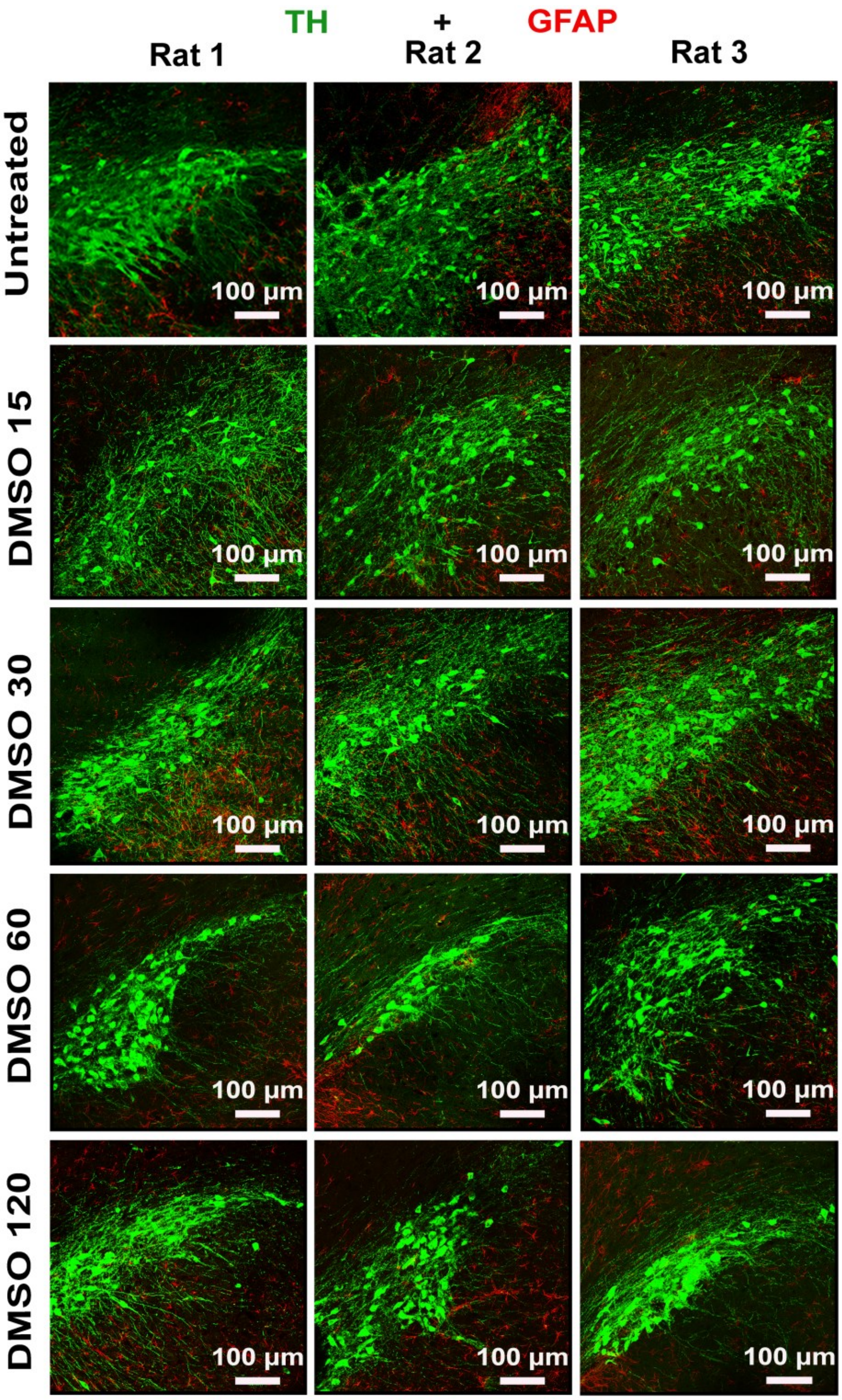

Set 2: DMSO effect on dopaminergic neurons (TH) and astrocytes (GFAP) compared with untreated controls. Quantification data appear in the graph of Figure 4(b) for TH and Figure 4(c) for GFAP.

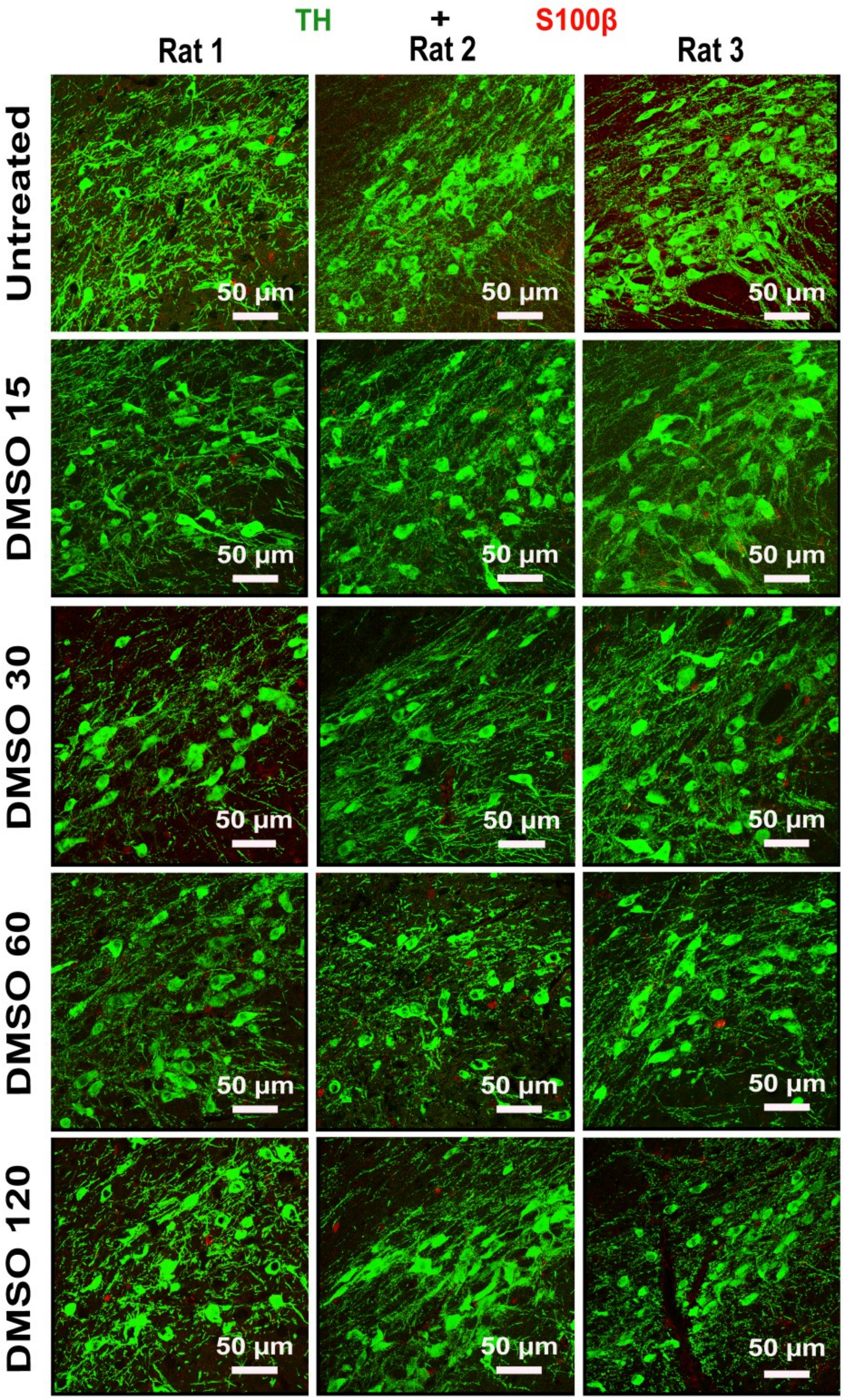

Set 3: DMSO effect on dopaminergic neurons (TH) and astrocytes (S100β) compared with untreated controls. Quantification data appear in the graph of Figure 5(b) for TH and Figure 5(b) for S100β.

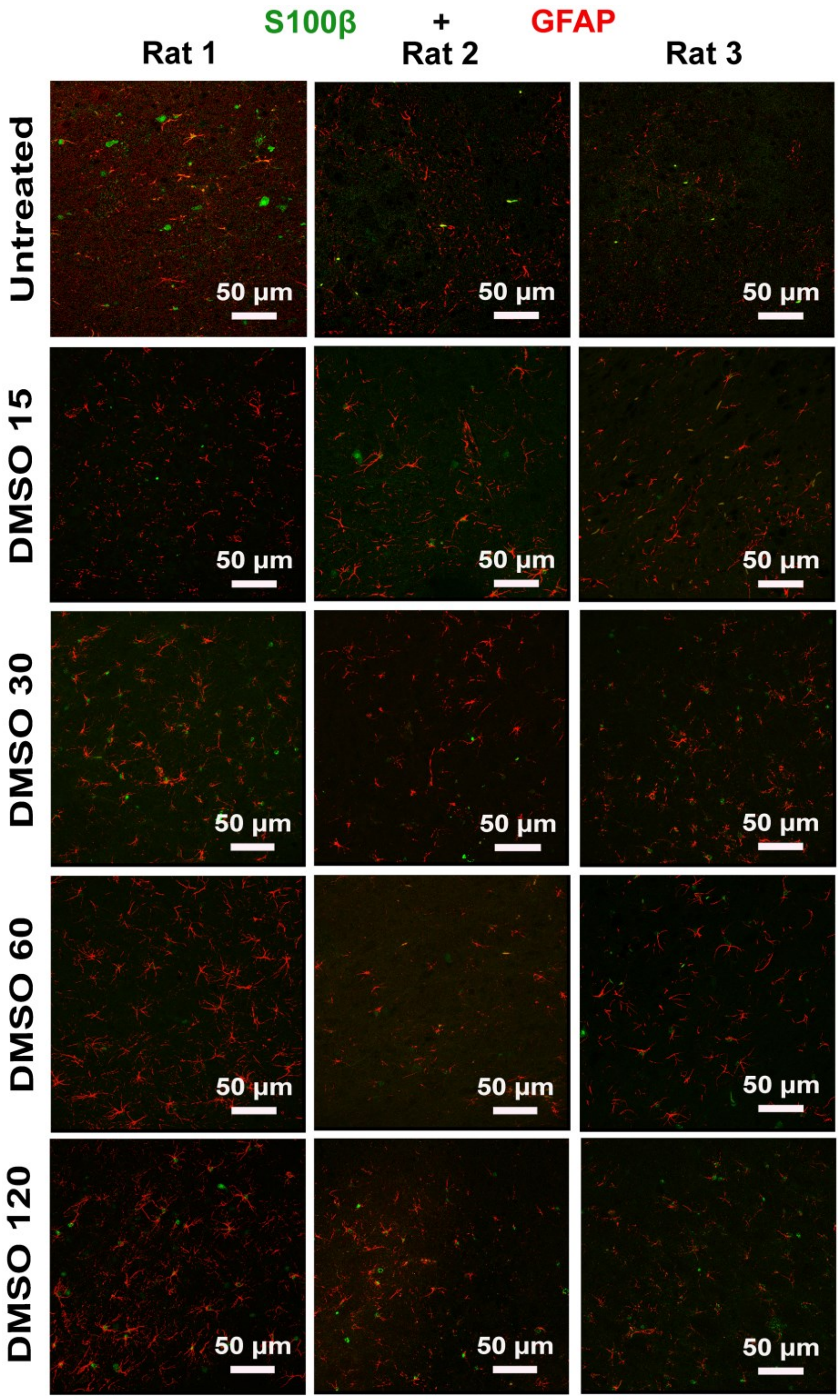

Set 4: DMSO effect on astrocytes (S100β) and (GFAP) compared with untreated controls. Quantification data appear in the graph of Figure 6(b) for S100β and Figure 6(c) for GFAP.

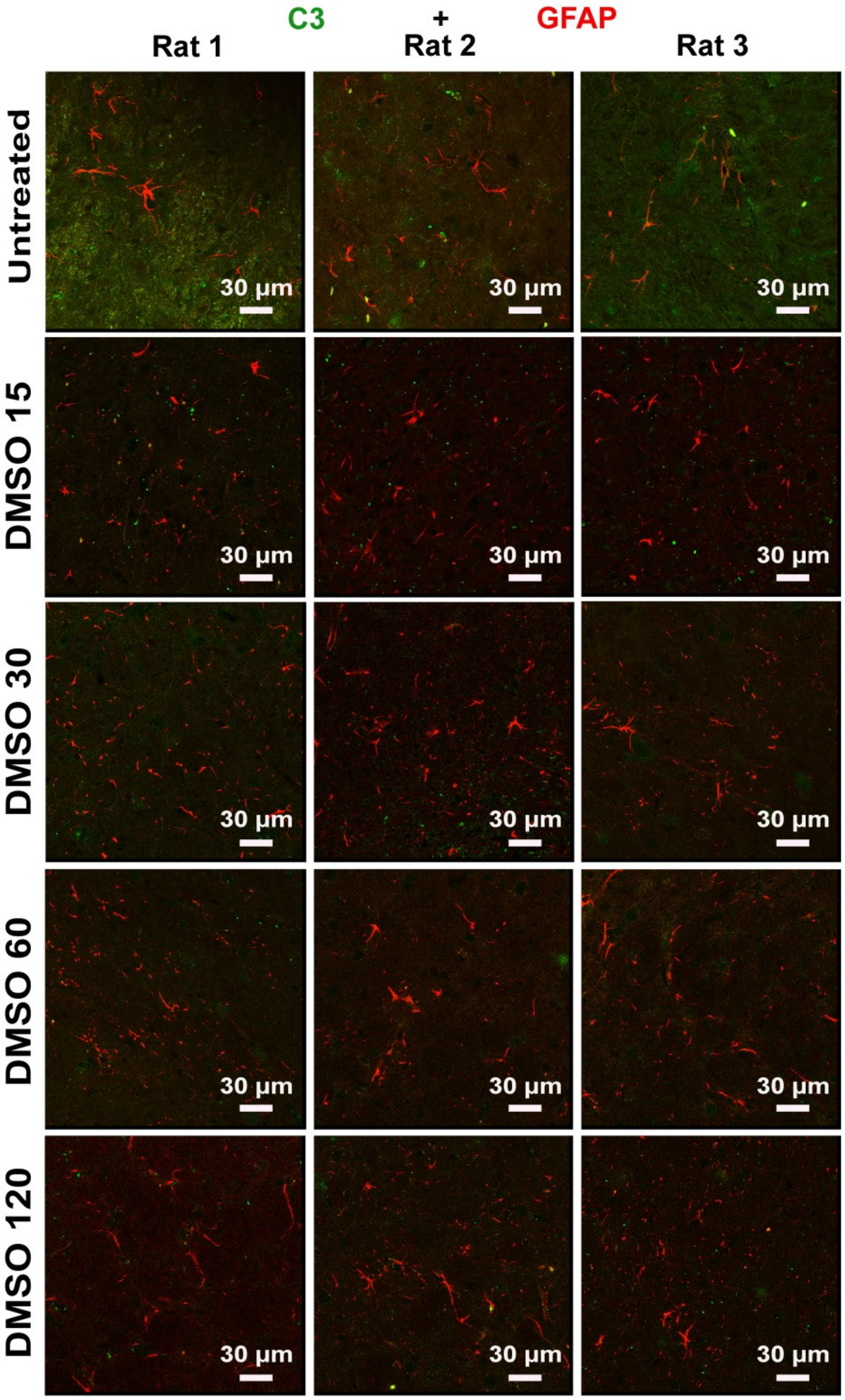

Set 5: DMSO effect on astrocytes (C3) and (GFAP) compared with untreated controls. Quantification data appear in the graph of Figure 7(b) for C3 and Figure 7(c) for GFAP.

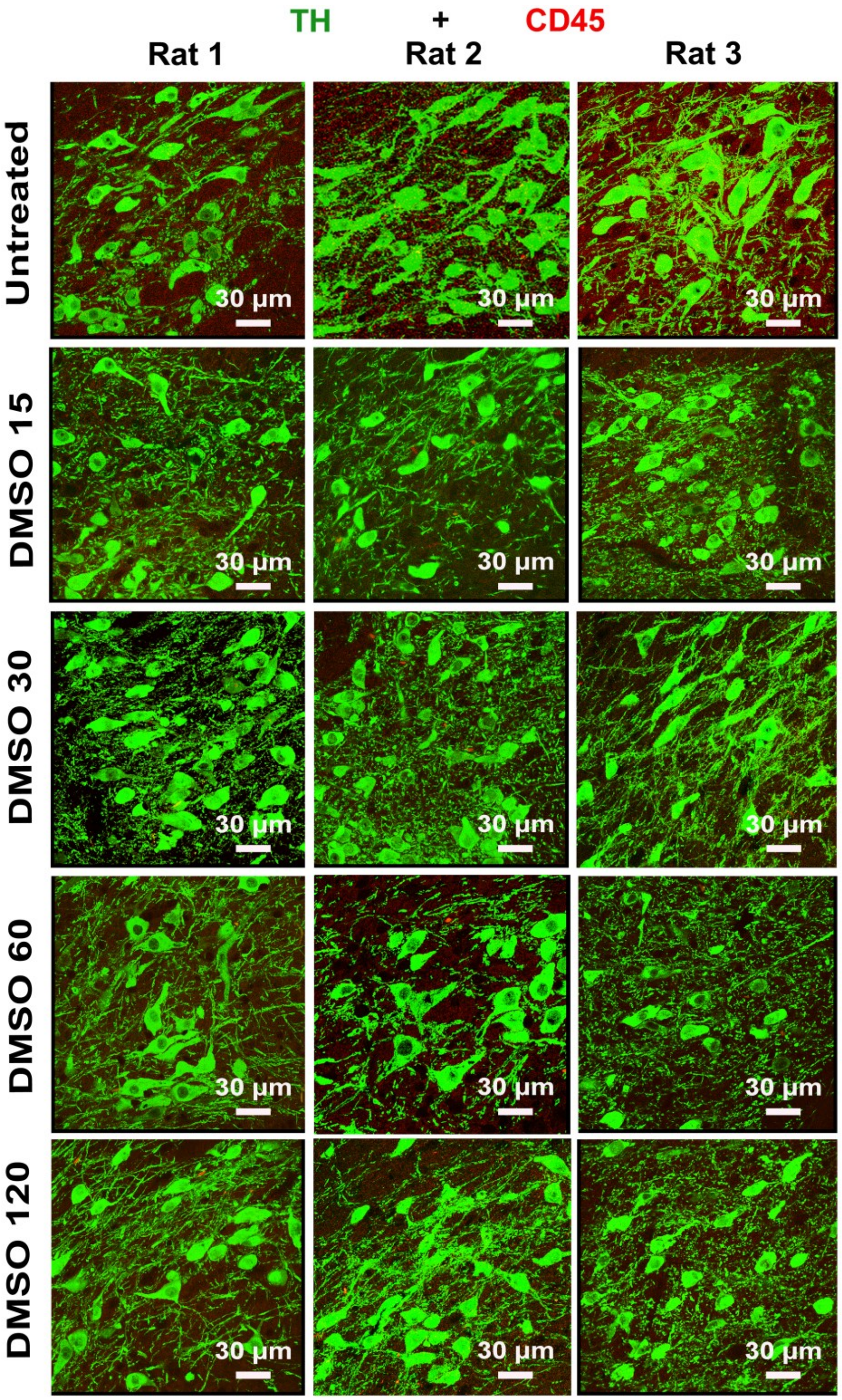

Set 6: DMSO effect on dopaminergic neurons (TH) and leukocytes (CD45) compared with untreated controls. Quantification data appear in the graph of Figure 9(b) for TH and Figure 9(c) for CD45.

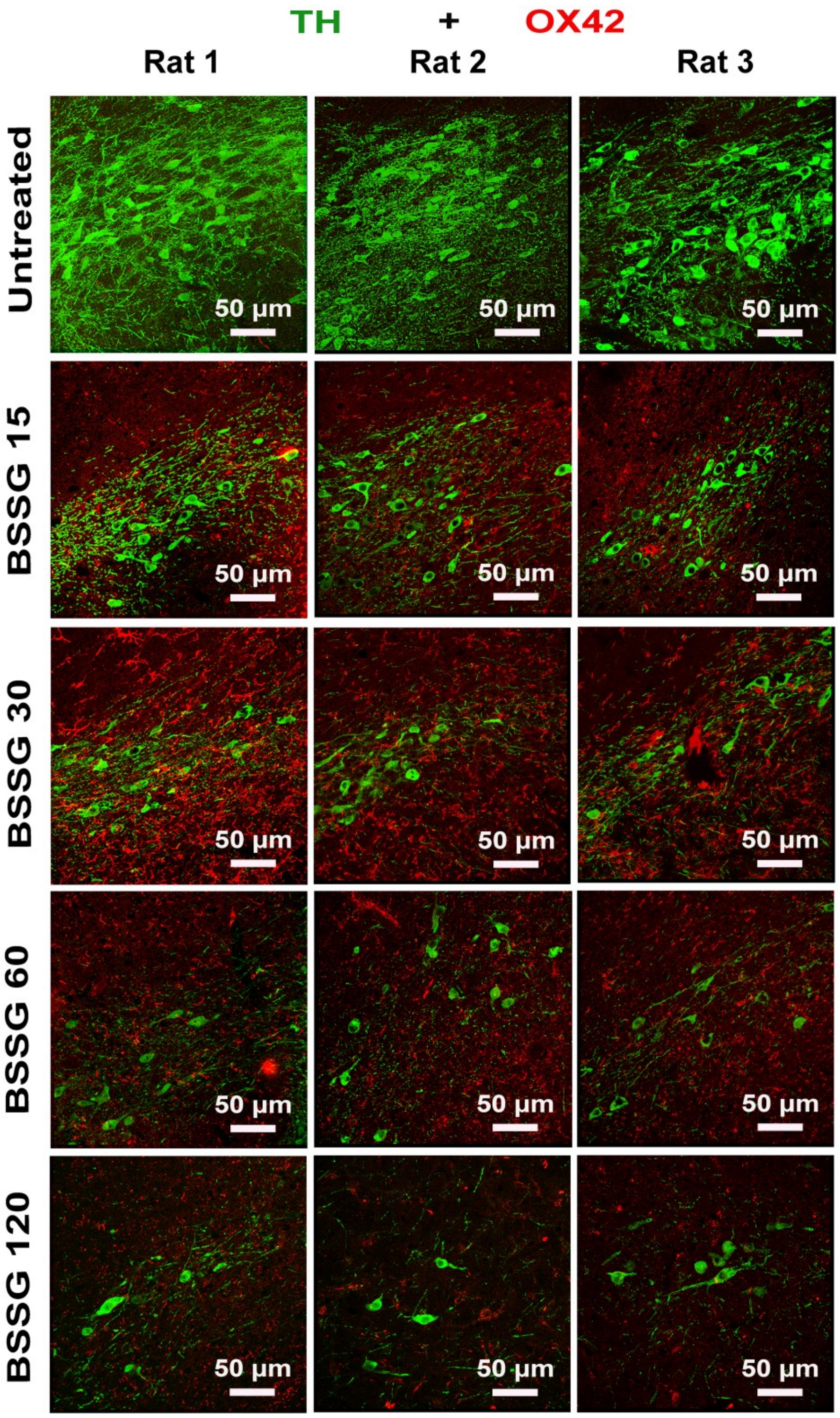

Set 7: BSSG effect on dopaminergic neurons (TH) and microglia (OX42) compared with untreated controls. Quantification data appear in the graph of Figure 2(b) for TH and Figure 2(c) for OX42.

for GFAP.

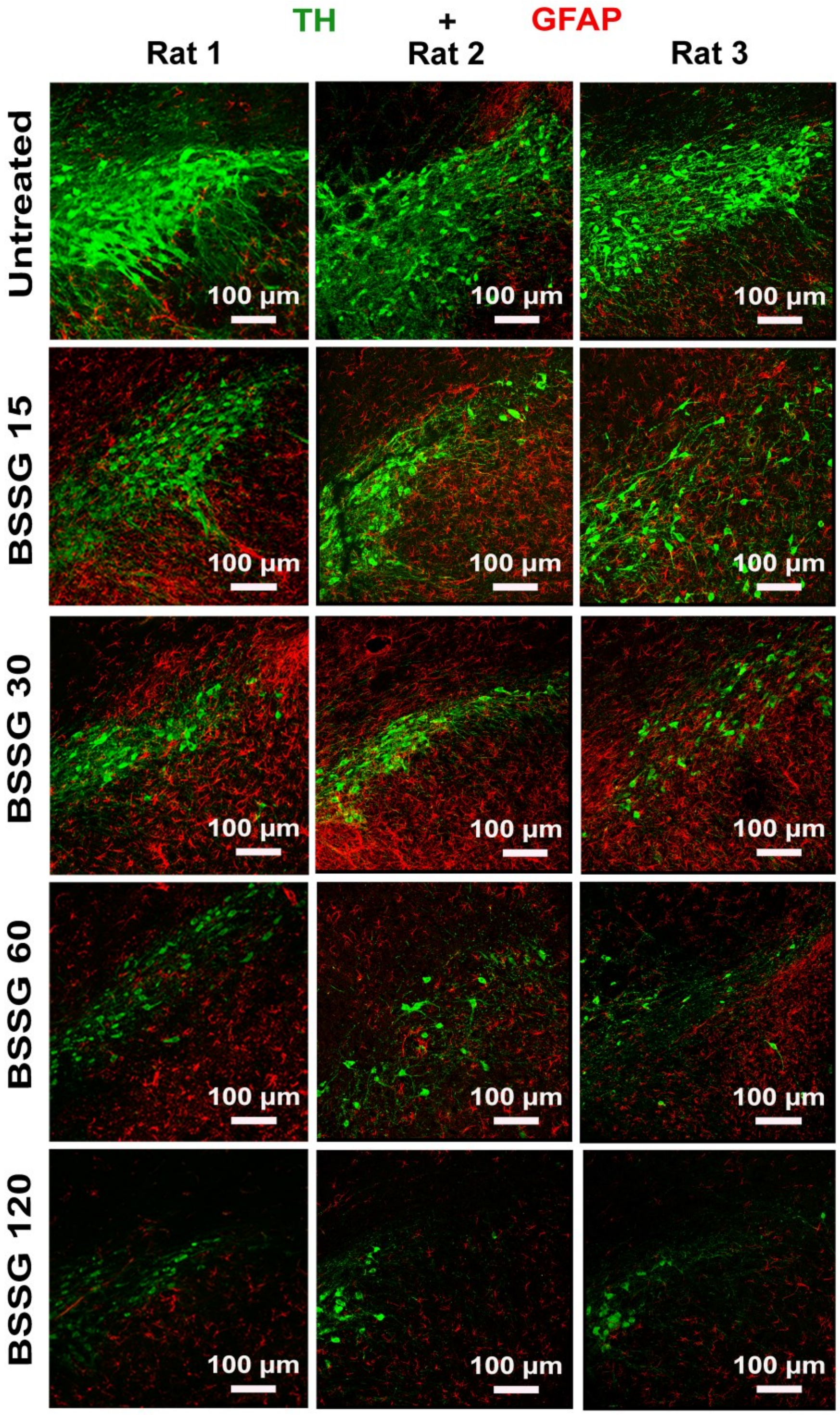

Set 8: BSSG effect on dopaminergic neurons (TH) and astrocytes (GFAP) compared with untreated controls. Quantification data appear in the graph of Figure 4(b) for TH and Figure 4(b) for GFAP.

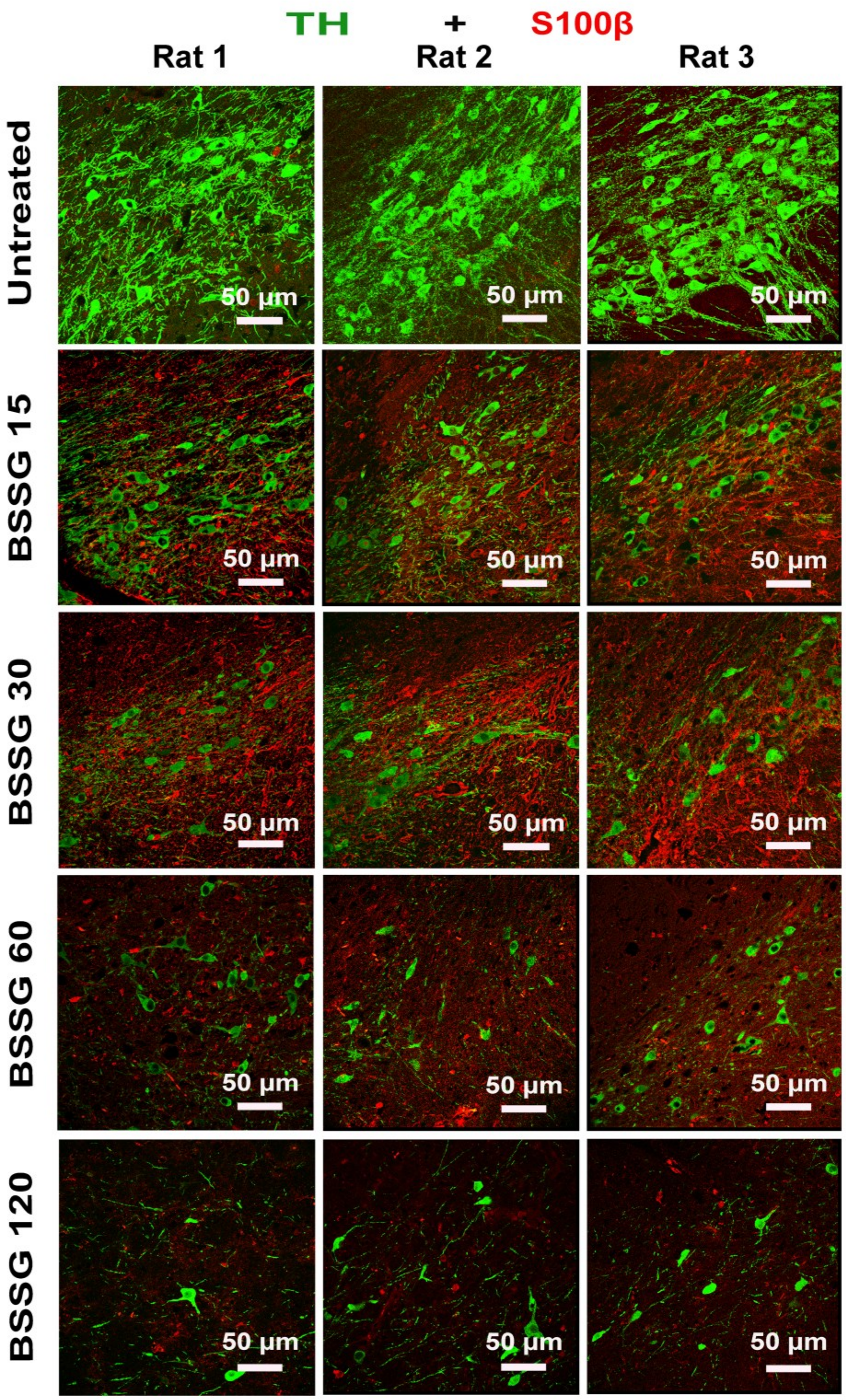

Set 9: BSSG effect on dopaminergic neurons (TH) and astrocytes (S100β) compared with untreated controls. Quantification data appear in the graph of Figure 5(b) for TH and Figure 5(b) for S100β.

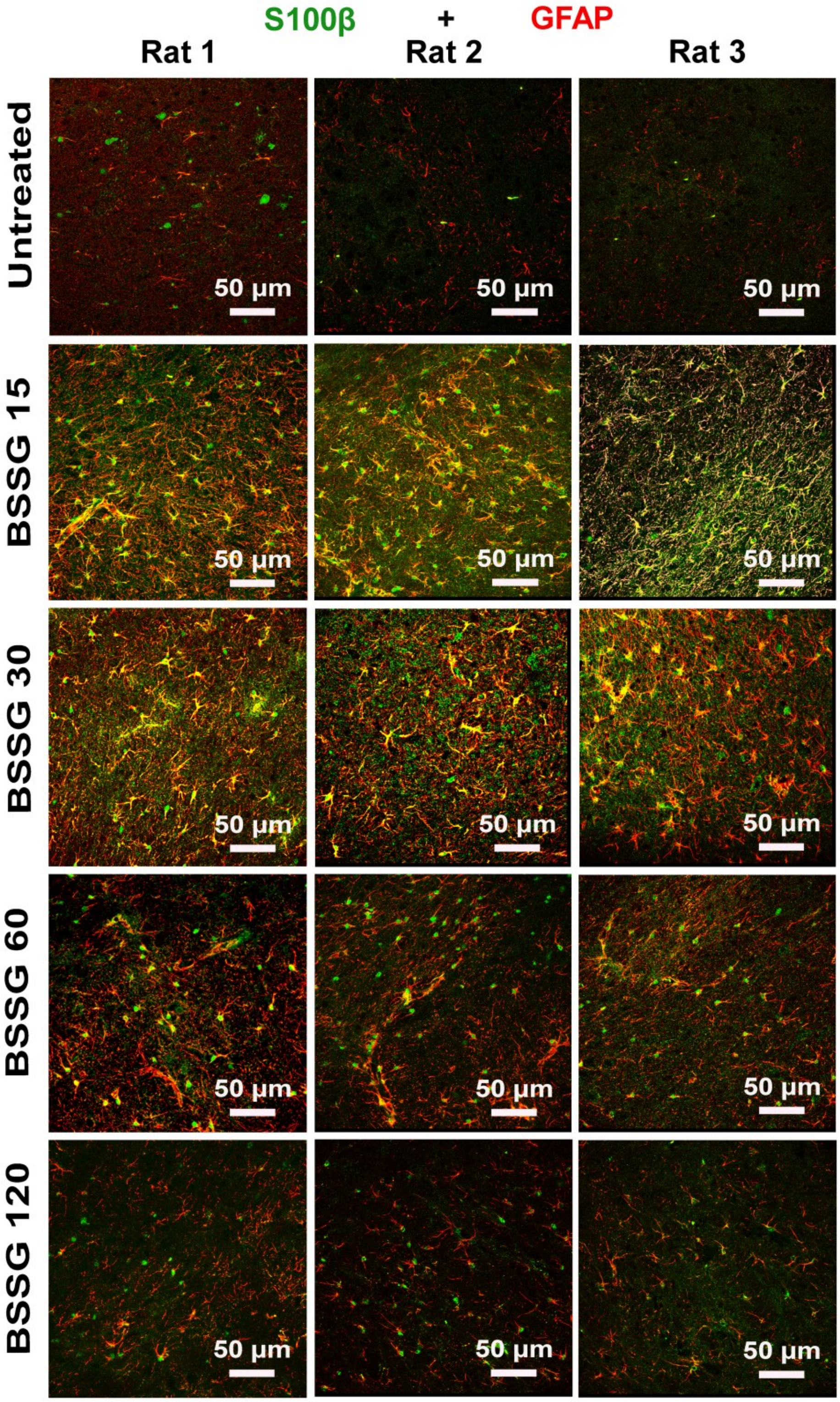

Set 10: BSSG effect on astrocytes (S100β) and (GFAP) compared with untreated controls. Quantification data appear in the graph of Figure 6(b) for S100β and Figure 6(c) for GFAP.

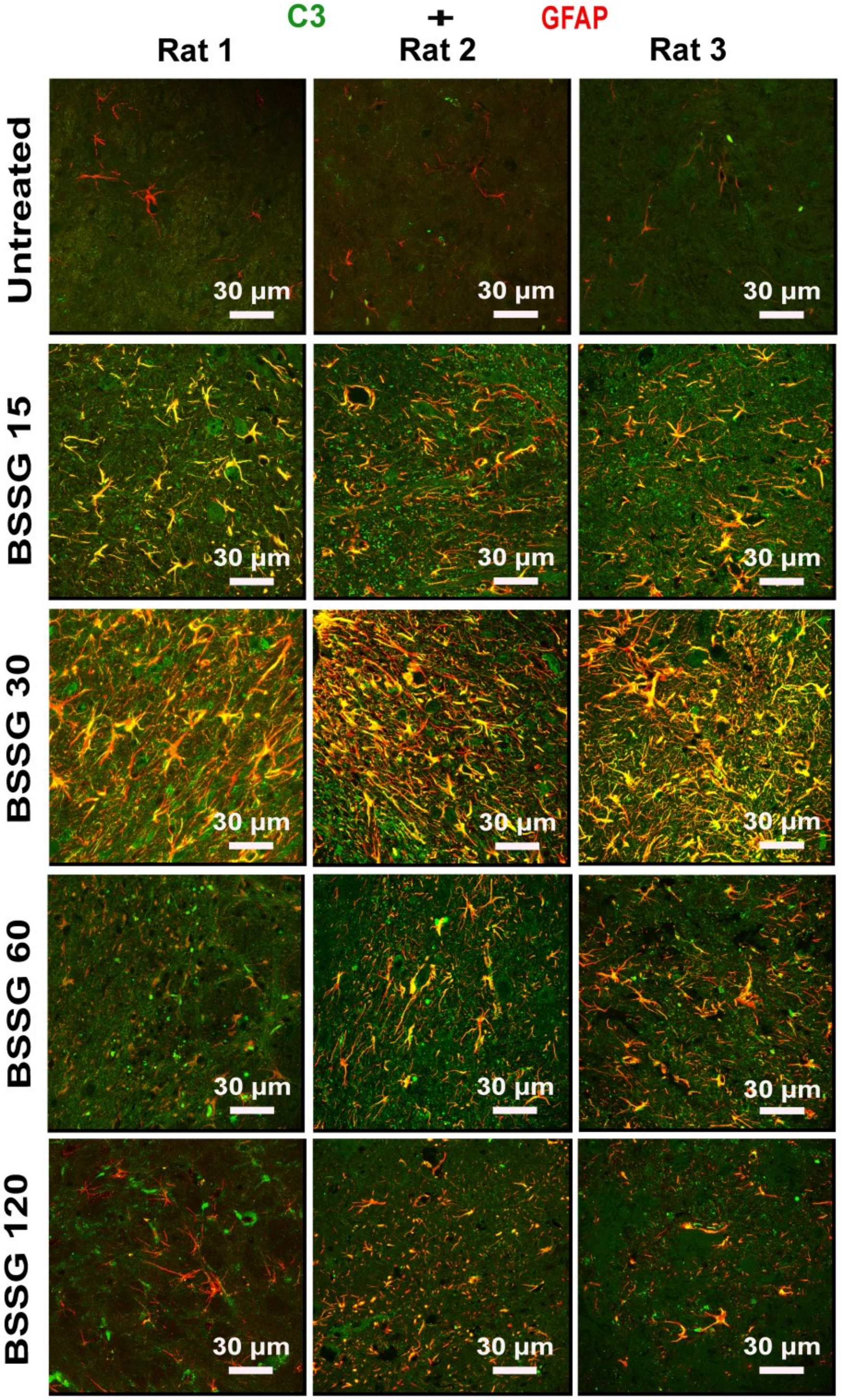

Set 11: BSSG effect on astrocytes (C3) and (GFAP) compared with untreated controls. Quantification data appear in the graph of Figure 7(b) for C3 and Figure 7(c) for GFAP.

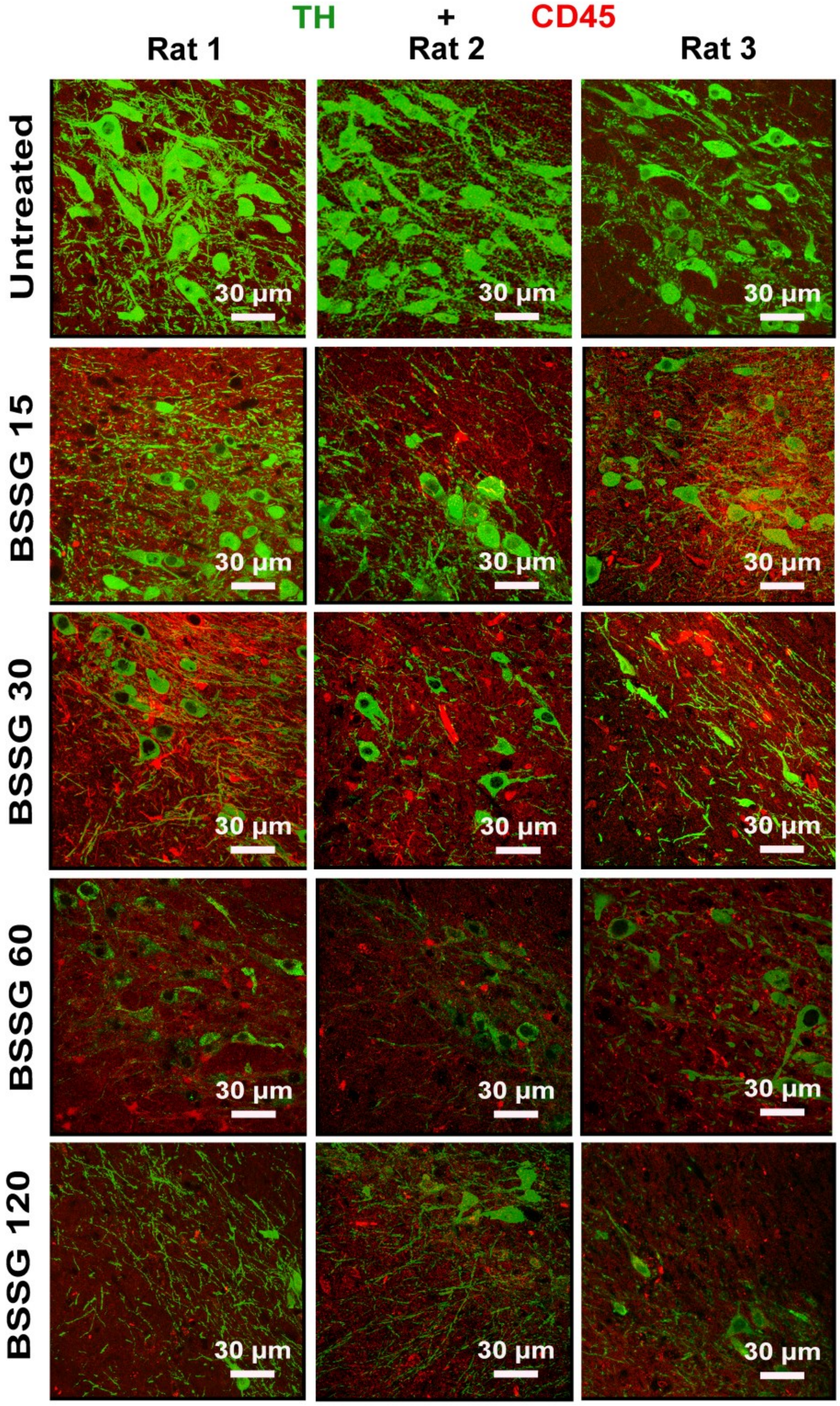

Set 12: BSSG effect on dopaminergic neurons (TH) and leukocytes (CD45) compared with untreated controls. Quantification data appear in the graph of Figure 9(b) for TH and Figure 9(c) for CD45.
